# From Pixel Modification to Generative Synthesis: A Survey of Deep Learning for Image Data Hiding

**DOI:** 10.3390/jimaging12090417

**Published:** 2026-09-04

**Authors:** Matúš Janok, Radoslav Forgáč, Ladislav Hluchý

**Affiliations:** Institute of Informatics, Slovak Academy of Sciences, 845 07 Bratislava, Slovakia; radoslav.forgac@savba.sk (R.F.); ladislav.hluchy@savba.sk (L.H.)

**Keywords:** data hiding, deep generative steganography, image steganography, image watermarking, proactive watermarking, zero-watermarking

## Abstract

This survey presents a structured review of deep learning-based techniques for image data hiding, proposing a three-paradigm taxonomy organized by the method’s operational relationship to the carrier image. We classify existing methods into modification-based, synthesis-based, and logic-based approaches. In the modification-based tier, we trace the architectural progression from foundational Convolutional Neural Networks and Generative Adversarial Networks to high-capacity Invertible Neural Networks and Transformers, analyzing their distinct trade-offs between embedding capacity, imperceptibility, and robustness. In the synthesis-based tier, we examine how Diffusion Probabilistic Models and generative adversarial frameworks reframe data hiding as a carrier generation problem rather than a pixel editing task. This paradigm encompasses both generative steganography (where carriers are synthesized from scratch) and proactive watermarking (where provenance is embedded during AI content generation). In the logic-based tier, we review zero-watermarking and coverless steganography, where ownership is established through feature extraction and semantic mapping without modifying any image, a critical property for sensitive domains such as medical imaging. Finally, we identify four persistent infrastructure gaps: benchmarking fragmentation, narrow robustness evaluation, domain generalization failures, and computational infeasibility that prevent real-world deployment despite architectural progress, and we propose concrete research directions.

## 1. Introduction

The volume of image content being transmitted across digital space is growing at a substantial and accelerating rate, extending beyond social media to encompass high-value assets such as digital art, satellite imagery, and confidential medical records. As these critical images are increasingly transmitted across public and private networks, protecting them against unauthorized access, illegal redistribution, and copyright infringement has become a significant challenge, particularly in the healthcare sector [1]. Digital images are inherently vulnerable due to their high dimensionality and perceptual redundancy, creating a pressing need for techniques that protect content integrity while preventing the creation and misuse of hidden channels.

This challenge is further amplified by growing concerns about digital content authenticity. Contemporary generative models enable the creation of hyperrealistic deepfakes, where subtle content manipulations can fuel disinformation campaigns and identity theft [2]. At the same time, the large size of an image makes it an ideal container for covert operations. Malicious actors can exploit this to construct hidden channels for malware delivery, confidential data exfiltration, covert communication [3], or other emerging threats like advanced backdoor attacks [4,5].

Cryptography, although foundational for data security, is inherently unsuited to covert media protection. Encryption renders content unreadable, thereby advertising the presence of hidden information rather than concealing it. Furthermore, naive block ciphers (ECB mode) applied directly to images produce ciphertext with detectable statistical regularities that expose encrypted payloads to statistical cryptanalysis [6]. Modern high-security frameworks therefore increasingly integrate chaotic systems with cryptographic primitives to improve unpredictability [7], though the fundamental visibility of encrypted media remains unresolved.

Data hiding addresses precisely this gap. Rather than rendering content unreadable, it operates beneath the threshold of perception. Digital watermarking embeds a persistent signature for copyright protection and tamper detection, prioritizing robustness against manipulation [8]. Steganography goes further, concealing not just the content of a message but its very existence, enabling covert communication without raising any suspicion [9].

### 1.1. Existing Surveys and Contributions

Despite the growing number of reviews in the literature, existing surveys primarily organize methods by architectural choice or application domain rather than operational intent. Among the general deep learning data hiding surveys, Wang et al. [10] provided a unified view of watermarking and steganography organized by architecture (CNN, GAN) and noise injection strategy, though without coverage of attention-based or diffusion approaches. Hosny et al. [11] surveyed deep learning-based image watermarking by network type (GAN, DNN, and CNN), providing a statistical analysis of model usage trends, but restricted their scope to watermarking only. Hu et al. [12] offered a more comprehensive review organized along three axes: neural network type (DNN, CNN, GAN, and INN), model structure design, and training strategy. Whereas their inclusion of INNs as a first-class paradigm is a notable contribution, their taxonomy remains rooted in modification-based frameworks, with diffusion-based and logic-based approaches left outside its scope. Dzhanashia and Evsutin [13] organized the field specifically by the role neural networks play within the embedding pipeline, providing quantitative comparisons of imperceptibility, capacity, and robustness across selected methods. Zhong et al. [14] similarly focused on deep learning-based watermarking, proposing a taxonomy of three categories: embedder-extractor, deep networks for feature transformation, and hybrid methods. Other researchers have focused on specific application domains: Priyadarshini et al. [15] surveyed watermarking techniques specific to medical imaging, whereas Nguyen et al. [16] approached the field from a copyright protection standpoint, organizing methods by technology layer (digital watermarking, deep learning, and blockchain). Wan et al. [17] surveyed robust image watermarking across both traditional and learning-based methods, and Sharma et al. [18] surveyed image watermarking through the lens of identity protection, covering over 100 works using a two-phase taxonomy based on embedding domain and watermarking attributes.

The common limitation of the existing surveys is that they primarily organize data-hiding methods by the architecture used or the application domain, rather than by the operational relationship established with the carrier image. This can group methods that share an architecture but pursue fundamentally different objectives. For example, Diffusion Probabilistic Models (DPMs) support both generative steganography, which synthesizes carriers to conceal payloads (CRoSS [19], VINE [20]), and proactive watermarking, which embeds provenance signals during content generation (Tree-Ring [21], The Stable Signature [22]). Likewise, GANs appear in all three operational roles of our taxonomy: modifying existing covers (HiDDeN [23]), synthesizing carriers (Volkhonskiy et al. [24], S-CycleGAN [25]), and extracting features for zero-watermarking (Res-GAN [26]). These examples show that architecture is orthogonal to operational intent. Moreover, zero-watermarking and coverless steganography do not directly modify or synthesize a carrier, making their defining characteristics difficult to express through a network-centric classification. Our taxonomy instead groups methods by what they operationally do with the carrier: modify it, synthesize it, or establish a logical association, providing a unified and architecture-independent basis for comparison.

To the best of our knowledge, none of the existing surveys on deep learning-based image data hiding provides a unified view that bridges the full operational spectrum from pixel-level modification through generative synthesis to logic-based verification without any image alteration. A comparison of these surveys is presented in Table 1.

This survey makes two primary contributions:

1. Three-Paradigm Operational Taxonomy. We classify methods by their relationship to the carrier image: modification-based (altering existing images), synthesis-based (generating carriers during hiding), and logic-based (establishing ownership through features without modification). This reveals that architectural evolution has been driven by paradigm-specific constraints rather than universal optimization.
2. Architectural Evolution Analysis. We demonstrate that each major architectural transition from Convolutional Neural Networks (CNNs), through GANs, Invertible Neural Networks (INNs), Transformers to diffusion models addressed a predecessor’s limitation while introducing new constraints, establishing that the robustness-capacity-imperceptibility trade-off reflects information-theoretic boundaries that no single architecture can circumvent.

### 1.2. Survey Methodology and Selection Criteria

To provide a comprehensive and technically focused overview of the evolution of deep-learning-based image data hiding, a structured literature search was conducted using IEEE Xplore, ScienceDirect (Elsevier), and SpringerLink. In addition, arXiv was searched to identify relevant preprints, technical reports, and recent research that may not yet have been indexed in the aforementioned digital libraries. The search covered publications from 2017 to early 2026. The search strategy combined terms related to the target applications, including “Image Data Hiding,” “Image Steganography,” and “Image Watermarking,” combined with auxiliary terms including “Machine Learning Steganography,” “Deep Learning Watermarking,” “Deep Generative Steganography,” and “Machine Learning Data Hiding.”

The study selection followed predefined inclusion and exclusion criteria, with an emphasis on deep-learning-based methodological and architectural contributions. Papers were considered eligible when they addressed image steganography, image watermarking, or image data hiding using deep neural networks, deep generative models, or related deep-learning architectures, and when they introduced a substantive methodological or architectural contribution. In particular, priority was given to works that established new deep-learning paradigms, introduced structurally novel architectures, formulated new embedding or extraction mechanisms, or enabled capabilities that could not be readily obtained through incremental optimization of established deep-learning methods. Classical machine-learning approaches without a deep-learning component were outside the scope of this survey. Papers were also excluded when they were unrelated to image data hiding, duplicated another identified publication, focused primarily on application-specific deployment without a substantive methodological contribution, or introduced only minor modifications, parameter tuning, or performance optimization of an established architecture.

**Figure 1:**
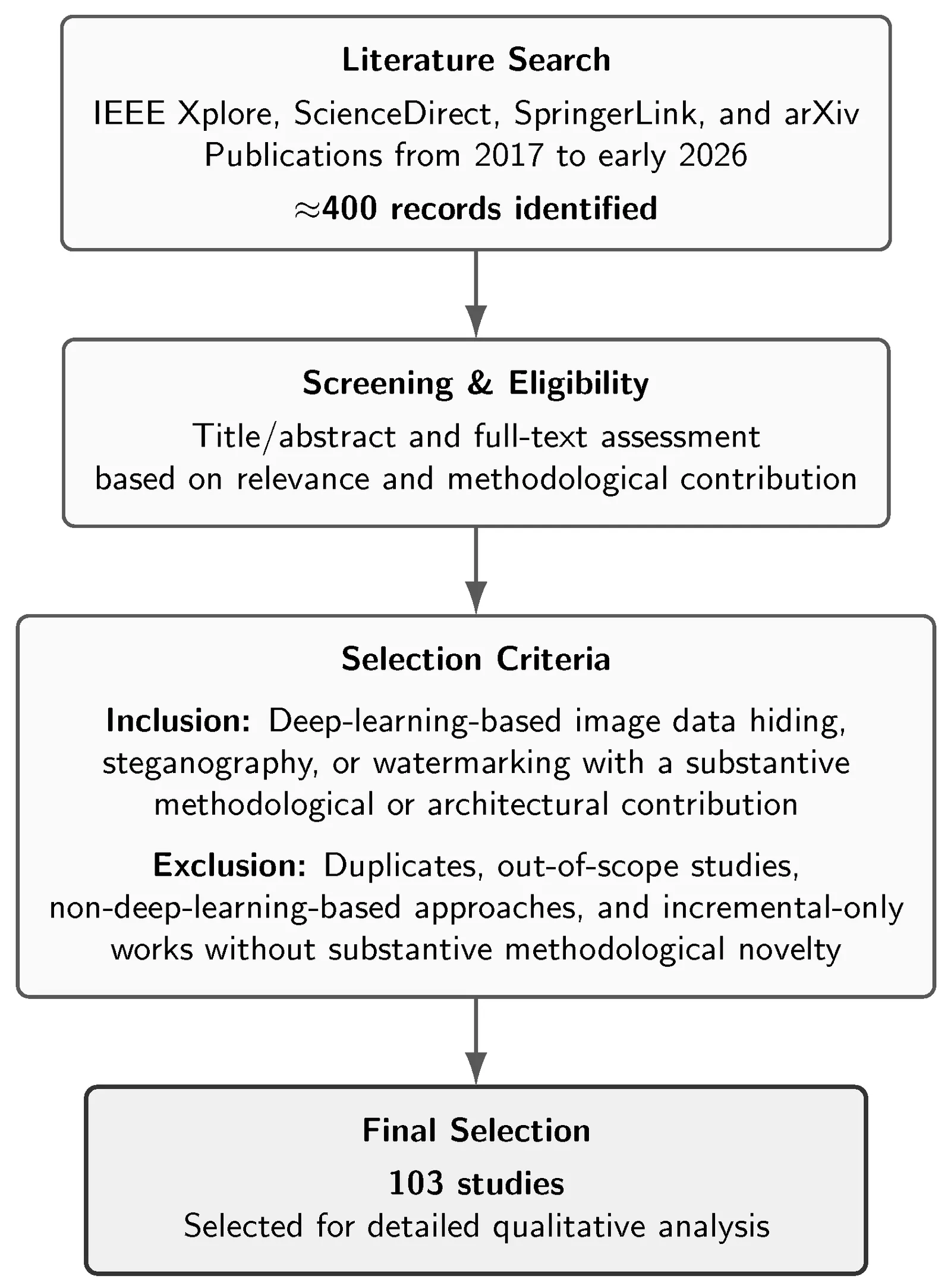
Literature search and study selection process. The search covered IEEE Xplore, ScienceDirect, SpringerLink, and arXiv for publications from 2017 to early 2026. Approximately 400 candidate publications were identified and screened according to predefined inclusion and exclusion criteria focused on deep-learning-based image data hiding and substantive methodological or architectural contributions. A final set of 103 studies was selected for detailed analysis.

The initial search yielded approximately 400 candidate publications. Following duplicate removal and screening of titles and abstracts, potentially relevant studies were examined for eligibility based on their relevance to deep-learning-based image data hiding and their methodological or architectural contribution. Publications that did not satisfy the predefined criteria were excluded during this process. The final selection comprised 103 studies for detailed analysis. The overall selection procedure is summarized in the flow diagram shown in Figure 1.

The survey proceeds as follows. Section 2 establishes the foundational terminology, evaluation metrics, and attack taxonomy. Section 3 systematically reviews architectures across the three paradigms: modification-based (CNNs, GANs, INNs, Transformers), synthesis-based (generative steganography and proactive watermarking), and logic-based (zero-watermarking and coverless methods). Section 4 identifies four open challenges: robustness evaluation, domain generalization, computational feasibility, and benchmarking fragmentation. Section 5 concludes the survey.

## 2. Foundations and Evaluation Methodology

A systematic analysis of image data hiding requires a clear definition of its underlying principles and operational constraints. This section outlines the fundamental terminology, defines the critical trade-offs inherent to the field, and categorizes existing techniques based on their embedding domains and robustness criteria.

### 2.1. Problem Formulation

**Figure 2:**
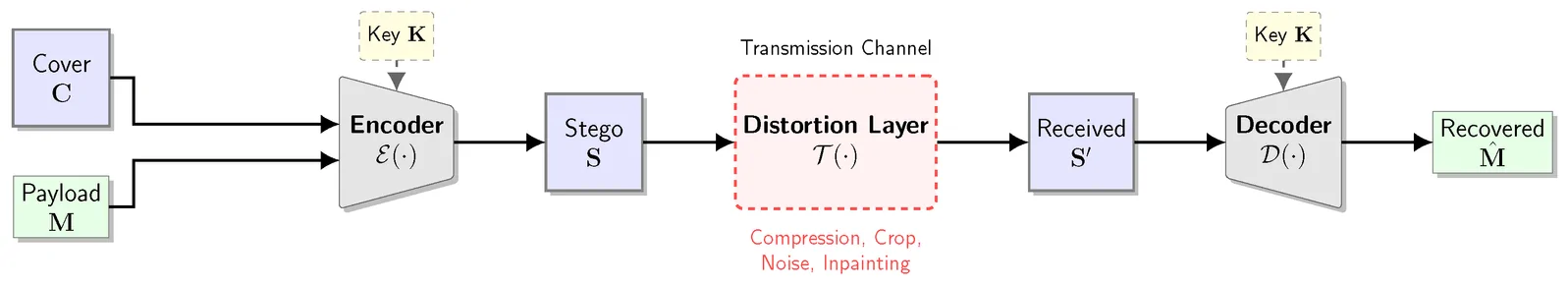
General framework for image data hiding. The encoder E embeds payload M into cover image C using secret key K, producing stego image S. During transmission, the noise layer T simulates various attacks (JPEG compression, cropping, AI-Generated Content (AIGC) editing, additive noise). The decoder D extracts the recovered payload $\hat{M}$ from the received image $S^{\prime }$ using the same key K.

The general framework of image data hiding is modeled as a communication channel, as illustrated in Figure 2. The process begins with the cover image C, which serves as the carrier for the payload M (the secret message or watermark). In deep learning-based frameworks, the key K may be incorporated as an explicit auxiliary input to the network or embedded implicitly into the model weights during training.

The embedding function E maps the cover C, the payload M, and an optional key K to a stego image S, formally defined as:

(1) S=E(C,M,K).

The generated S must perceptually resemble C as closely as possible. During transmission through a channel T, the image may undergo incidental or adversarial distortions (e.g., compression, noise), resulting in a received image $S^{\prime }=T\left(S\right)$. The decoding function D then attempts to reconstruct the payload, denoted as:

(2) $$\hat{M}=D\left(S^{\prime },K\right).$$

Ideally, $\hat{M}\approx M$ for robust watermarking, or $\hat{M}=M$ for lossless steganography.

### 2.2. Objectives and Trade-Offs

**Figure 3:**
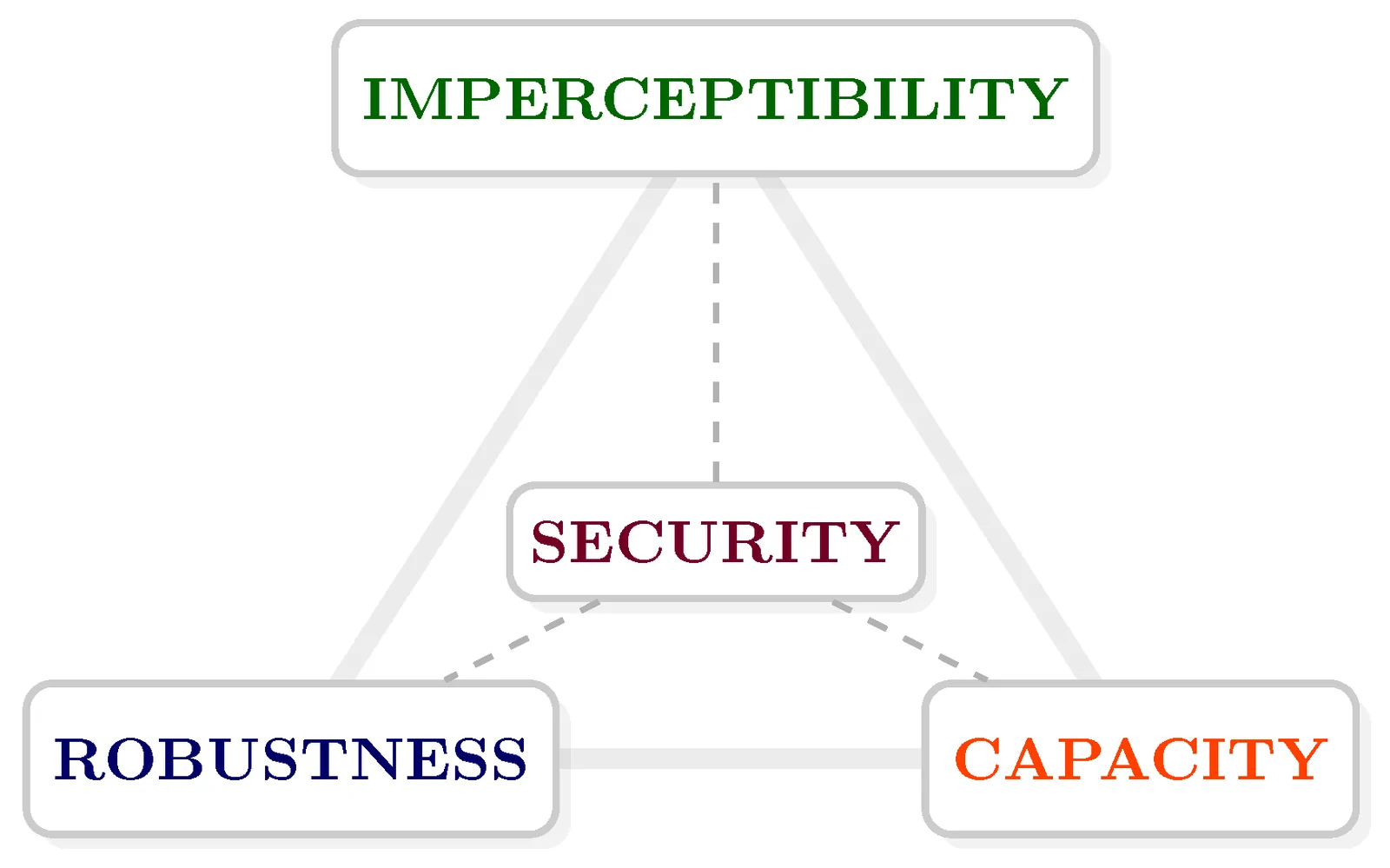
Trade-off triangle of core objectives in image data hiding. Optimizing any single objective typically degrades one or both of the others. Security (center) is an orthogonal constraint that interacts with all three vertices simultaneously.

The efficacy of data hiding is evaluated against three primary objectives and one orthogonal security constraint, forming a trade-off triangle (Figure 3) where optimizing one parameter often degrades the others.

Robustness measures the resilience of the embedded data against distortions. It is the primary requirement for copyright protection, where the watermark must survive malicious attacks (e.g., cropping, blurring) and benign processing (e.g., JPEG compression) to remain detectable.

Imperceptibility quantifies the perceptual similarity between the cover C and the stego image S. High imperceptibility is crucial, as visible artifacts would defeat the very purpose of data hiding. Effective data hiding methods take advantage of the Human Visual System’s (HVS) non-uniform sensitivity by embedding information in regions where distortions are less likely to be noticed, typically textured or high-frequency areas. To ensure both visual imperceptibility and resilience to such distortions, it is beneficial to adapt the embedding strategy by emphasizing specific regions of the image [13]. To quantify this, Just Noticeable Difference (JND) models are often employed to estimate the visual thresholds of human perception [27]. These models account for luminance and texture masking effects in different image regions, ensuring that modifications remain below the threshold of human perception [28].

Capacity denotes the maximum information volume (in bits per pixel, bpp) that can be embedded. Increasing capacity typically introduces greater distortion (lowering imperceptibility) or fragility (lowering robustness). To mitigate bit errors in high-capacity regimes, Error Correction Codes (ECC) such as Reed-Solomon are frequently integrated [29].

Security is a critical objective and it refers to the system’s resistance to detection by an active adversary or steganalysis algorithm. A secure method ensures that the statistical distribution of S is indistinguishable from C, preventing an attacker from determining whether hidden data exists, even if they cannot decode it.

### 2.3. Steganography vs. Watermarking

Although steganography and watermarking share common architectural foundations, typically built on encoder–decoder principles, their operational objectives diverge significantly, dictating different trade-offs among the objectives defined above.

Steganography prioritizes undetectability through a trade-off between imperceptibility and capacity. The primary goal is to establish a covert communication channel where the mere existence of the message remains statistically invisible to steganalysis tools. In such scenarios, robustness against distortion is often secondary to maintaining the statistical integrity of the cover image.

Watermarking, conversely, focuses on robustness and provenance. Here, the presence of the hidden signal is often assumed or public and the critical challenge is ensuring the mark remains irremovable and detectable despite malicious attacks or benign processing. Capacity is typically low (e.g., a 64-bit identifier) compared to steganography.

Throughout this survey, we organize methods by their operational paradigm while explicitly noting whether specific architectures are tailored to meet the high-capacity demands of steganography or the resilience requirements of watermarking.

### 2.4. Embedding Domains

Image hiding methods can be broadly categorized according to the domain in which the payload is embedded. This choice fundamentally influences key system properties, including robustness, imperceptibility, payload capacity, and computational complexity.

#### 2.4.1. Spatial Domain

Spatial domain methods embed information by directly modifying the pixel intensities of the cover image. The classic example is Least Significant Bit (LSB) substitution [30,31], in which payload bits replace the lowest bit-planes of pixel values. Whereas LSB-based approaches are computationally efficient and provide high embedding capacity, they are highly sensitive to common signal processing operations and vulnerable to statistical steganalysis [32].

To mitigate these limitations, more advanced spatial techniques have been proposed. Pixel Value Differencing (PVD) [33] exploits the reduced sensitivity of the human visual system in edge and texture regions to adaptively embed data with lower perceptual distortion. Histogram Shifting [34] modifies pixel intensity distributions to create reversible embedding space, enabling lossless recovery of the original image. Despite these improvements, spatial-domain methods remain fundamentally limited in robustness against lossy compression, filtering, and geometric distortions due to their direct manipulation of pixel-level representations.

#### 2.4.2. Transform Domain

Transform domain approaches embed information into frequency coefficients rather than directly altering pixel values. The transform acts as a pre-processing step that decomposes the image into components where embedded signals can achieve increased robustness against compression and common image processing operations.

Frequency and Multi-resolution Transforms

The Discrete Cosine Transform (DCT) [35] and Discrete Fourier Transform (DFT) [36] are the foundational algorithms for robust watermarking. The DCT is the standard basis for JPEG-robust watermarking due to its energy compaction properties, whereas the DFT enables geometric invariance through its magnitude spectrum. To address the lack of spatial localization in global transforms, the Discrete Wavelet Transform (DWT) [37] is frequently employed to split inputs into frequency sub-bands, allowing embedding in texture-rich areas.

Advanced Matrix and Hypercomplex Transforms

To capture complex geometric features that DWT misses, recent frameworks have integrated directional transforms like the Non-Subsampled Shearlet Transform (NSST) [38] and algebraic factorizations like Singular Value Decomposition (SVD) [39].

For color images, quaternion-domain representations have emerged as a specialized alternative. Conventional transforms typically process RGB channels independently, thereby neglecting inter-channel correlations. Quaternion-based transforms, such as the Quaternion Discrete Fourier Transform (QDFT) and Quaternion Wavelet Transform (QWT), encode color images as unified vector fields, preserving inter-channel spectral relationships. This holistic representation has been shown to improve robustness against color-space manipulations and chromatic distortions compared to scalar, channel-wise approaches [40,41].

#### 2.4.3. Latent Domain

The rapid advancement of deep learning has introduced learned representations as a fundamentally different embedding space, distinct from both spatial and transform domain approaches. Rather than operating on predetermined mathematical transforms, these methods embed the payload into representations learned directly from data. This category spans a spectrum from feature-guided methods that optimize embedding in pixel space via learned non-linear transforms, to generative methods that operate inside a true compressed or noise latent code that is structurally disconnected from pixel space.

#### 2.4.4. Feature Domain

Feature domain methods do not embed a payload into any image representation. Instead, robust and invariant features are extracted from the original content and used to construct a separate verification record, leaving the source image entirely unmodified. This domain is exclusive to logic-based approaches: zero-watermarking derives a verification key from deep feature maps, whereas coverless steganography establishes a deterministic mapping between image features and message segments. The embedding domain is therefore the feature space itself rather than any pixel, frequency, or latent representation of the carrier.

- Feature-Guided Pixel Embedding: Architectures like HiDDeN [23] learn an optimal non-linear transform that distributes the message across the entire feature map during the forward pass. Critically, the payload manifests as pixel-level perturbations in the output stego image; the network’s internal feature space guides where and how to embed, but the embedding domain itself remains the pixel space, improving resilience against complex distortions over handcrafted transforms.
- Generative Latent Embedding: In Latent Diffusion Models (LDMs) [42], watermarking is performed inside the latent noise space of a Variational Autoencoder (VAE), a compressed representation that is structurally disconnected from pixel space. The watermark is embedded into the generative process itself, ensuring it is intrinsic to the synthesized content rather than applied as a post hoc perturbation.

By operating on learned representations rather than explicit spatial or frequency components, these methods enable increased resilience to semantic-preserving transformations and aggressive compression, offering a flexible and powerful alternative to classical image hiding domains.

### 2.5. Robustness Criteria

Based on the required level of robustness, methods can be classified into three categories: fragile, semi-fragile, and robust [43]. Fragile methods [44,45] are designed for content authentication and have high sensitivity to modifications. Any change to the stego image, no matter how small, will prevent the correct extraction of the verification payload. They are mostly used for tamper detection. Semi-fragile methods [46,47] offer a compromise, tolerating incidental distortions like mild compression while detecting malicious alterations. Robust methods [48,49] are designed to resist a wide range of attacks, including compression, noise, and geometric transformations. This makes them suitable for applications like copyright protection, where the payload must remain intact despite redistribution or intentional attempt to disrupt the embedded data [8].

### 2.6. Extraction Requirements

Image data hiding methods can also be classified based on the information required during the extraction process. This classification includes blind, non-blind, and semi-blind methods. Non-blind methods [50,51] require the original, unaltered cover image to detect and extract the embedded watermark. Although this requirement allows for very robust watermarking schemes, it is also their primary drawback. In most real-world scenarios, the original image is not available at the time of extraction, strongly limiting the practicality of non-blind methods. Semi-blind methods [52,53] require auxiliary information, such as specific features of the original image, key or hash, to extract the embedded data. Blind methods [54,55] do not require any information about the original image for extraction. They are also often the most practical choice for applications such as copyright protection or content authentication [56].

### 2.7. Evaluation Methodology

Evaluating the performance of image data hiding methods requires standardized metrics to quantify the four core objectives: robustness, imperceptibility, capacity, and security. Table 2 provides a mathematical summary of the metrics used across the literature.

#### 2.7.1. Robustness and Data Recovery Metrics

Robustness measures the ability of the watermark to survive transmission and attacks. The most fundamental metric is the Bit Error Rate (BER), which calculates the ratio of incorrectly extracted bits to the total payload length. A BER of 0 indicates perfect recovery, whereas 0.5 represents total loss of information (random guessing).

For scenarios involving image-based watermarks (e.g., logo embedding) or zero-watermarking, the Normalized Cross-Correlation (NCC) is the preferred metric. Unlike pixel-wise error, NCC assesses the structural similarity between the original and extracted watermark signals and is robust to global linear changes in brightness or contrast [57].

#### 2.7.2. Imperceptibility and Perceptual Quality Assessment

Evaluating visual quality in deep learning-based data hiding requires a distinction between pixel-level fidelity and perceptual semantic consistency.

Pixel-Level Metrics: For modification-based methods (e.g., CNNs), the standard metrics are Peak Signal-to-Noise Ratio (PSNR) [58] and Structural Similarity Index Measure (SSIM) [59]. Whereas PSNR measures absolute pixel error, SSIM correlates more effectively with human perception by modeling changes in luminance, contrast, and structure.

Perceptual Metrics for Generative Models: In generative frameworks, the evaluation objective shifts from strict pixel-wise fidelity toward perceptual realism and semantic consistency. Consequently, traditional metrics such as PSNR and SSIM often prove inadequate as they do not capture the high-level synthesis quality. Instead, Learned Perceptual Image Patch Similarity (LPIPS) [60] is utilized to quantify distances within deep feature spaces (e.g., VGG or AlexNet), whereas the Fréchet Inception Distance (FID) [61] provides a statistical comparison between the distributions of generated stego images and real-world datasets. To address the limitations of reference-based evaluations, no-reference quality metrics like BRISQUE [62] are increasingly discussed to evaluate visual quality when a pristine cover image is unavailable, while CIE color difference metrics like CIEDE2000 [63] provide a uniform color-space evaluation to capture chromatic distortions that standard luminance-focused metrics fail to represent. These quantitative benchmarks can also be supplemented by subjective human evaluation to verify that embedded signals remain below the threshold of conscious perception in hyperrealistic outputs [64].

#### 2.7.3. Capacity Metrics

Capacity quantifies the information volume per carrier. The absolute payload is the total number of embedded bits, providing a clear indication of total volume. To allow standardized comparisons across varying image resolutions, Bits Per Pixel (bpp) is utilized as the primary normalized metric [65].

#### 2.7.4. Statistical Security and Steganalysis Evaluation

Security is defined by a method’s resistance to steganalysis, the detection of hidden information by an unauthorized adversary. Evaluation typically involves a binary classification scenario where a detector attempts to distinguish between clean cover images and stego images.

The standard metric for security is the Receiver Operating Characteristic (ROC) curve, which plots the True Positive Rate against the False Positive Rate. A perfectly secure method yields an Area Under the Curve (AUC) of 0.5 (random guessing), whereas an AUC approaching 1.0 indicates high detectability.

Steganalysis methodologies have advanced from classical statistical heuristics to sophisticated deep neural frameworks. Whereas foundational tools such as StegExpose [66], a statistical detector designed for LSB steganography in JPEG images, are still utilized for baseline statistical assessments, the current established benchmarks are dominated by deep convolutional architectures, most notably Xu-Net [67] and SRNet [68].

Historically, these models were designed for binary classification specifically distinguishing steganographic carriers from original cover images. However, the focus has recently shifted to forensic identification, which seeks to determine the specific algorithm used for stego image creation. A representative example is the generic recognition scheme proposed by Gao et al. [69], which leverages “Big Data matching” and perceptual hashing to approximate original covers. By feeding difference features into a ResNet50 enhanced with Pyramid Split Attention (PSA) and an ArcFace loss, their method achieves over 96% accuracy in distinguishing between six different adaptive steganography algorithms. For a detailed taxonomy of these evolving steganalysis and forensic tools, we refer the reader to the comprehensive review by Mittal et al. [70].

### 2.8. Common Attack Scenarios

A robust image data hiding method must withstand various types of attacks that intend to distort or remove the embedded data. Although traditionally categorized into image processing, geometric, and cross-media attacks [71], the rise in deep learning has introduced sophisticated threats like generative editing and statistical estimation.

Signal-Level Processing Attacks: These involve common manipulations that an image might undergo during transmission, whether intentionally or incidentally. These include noise addition (Gaussian, Salt-and-Pepper, Poisson), lossy compression (JPEG), frequency filtering (average, median, Gaussian), and contrast adjustments [72].

Geometric Attacks: These focus on spatial transformations that alter pixel coordinates without necessarily destroying the signal. However, operations such as rotation, scaling, cropping, and translation introduce de-synchronization between the embedded pattern and the decoder’s receptive field, often rendering the signal unrecoverable for models lacking global context awareness [72].

Cross-Media Attacks: These occur when content transitions between digital and physical domains, such as the screen-shooting attack, where capturing a digital display with a camera introduces complex, non-linear analog distortions such as moiré patterns or lens glare [71]. Similarly, print-and-scan operations induce color degradation and resolution loss.

Generative Attacks: These represent modern threats utilizing AI-Generated Content (AIGC) technologies. By employing generative models like diffusion models or VAEs, attackers can create high-quality forgeries or modifications that can effectively remove or alter embedded data. The most common attack in the reviewed literature is Stable Diffusion inpainting [42], though by 2026 the threat landscape has expanded to include commercial generative erasure tools and more capable latent diffusion architectures, representing an evaluation gap for future work.

Statistical Estimation and Collusion Attacks: Unlike random distortions, these exploit the statistical consistency of embedding patterns across multiple images. As demonstrated by Yang et al. [73], many deep watermarking models embed content-agnostic noise patterns. An adversary can estimate this fixed pattern by averaging the residuals of multiple watermarked images, subsequently subtracting it to remove the watermark or injecting it into clean images to forge ownership credentials.

### 2.9. Datasets

Standardized datasets are essential for training and benchmarking the objectives of image data hiding methods. The choice of dataset typically depends on whether the goal is general-purpose feature learning, security evaluation, or high-resolution images (Table 3).

General-Purpose Training Sets

To ensure models generalize across diverse textures and scenes, large-scale computer vision datasets are the standard. ImageNet [74], with over 14 million images, remains the primary choice for pre-training encoder–decoder networks. For models requiring dense object context, the COCO dataset [75] is widely used. Whereas the PascalVOC dataset [76] was extensively used in early hiding research, it is now rarely employed, having been largely superseded by the superior scale and diversity of COCO and ImageNet.

Resolution and Domain-Specific Sets

For applications requiring high-fidelity recovery, such as copyright protection of digital art, DIV2K [77] offers high-resolution (2K) images often used to test robustness against scaling. For low resolutions, CIFAR-10 [78] is occasionally used for initial prototyping, though its low resolution (32×32) limits its utility across most embedding domains. Finally, facial image datasets like CelebA-HQ [79] are standard for evaluating watermarking in the context of deepfake prevention and identity protection.

Steganalysis and Security Benchmarks

Security-oriented methods require datasets with controlled noise profiles. BOSSbase v1.01 [80] and the BOWS2 dataset [81] are the historical gold standards for spatial-domain steganography and steganalysis, with both providing collections of 10,000 uncompressed grayscale images. However, because they are limited to grayscale content, the ALASKA datasets (v1 and v2) [82] were later introduced to address the need for color steganalysis. These provide a diverse collection of high-quality RAW and JPEG images, serving as the modern benchmark for resisting deep steganalysis tools.

**Table 3:** Summary of standard datasets used in image data hiding research.

Dataset	Type/Content	Scale	Primary Application
ImageNet [74]	General Objects (RGB)	14 M	General-purpose training; robustness benchmarking.
COCO [75]	Object Detection (RGB)	330 k	Training models requiring complex scene context.
BOSSbase [80]	Uncompressed (Grayscale)	10 k	Standard for steganalysis and security evaluation.
ALASKA v2 [82]	High-Quality (RAW)	80 k	Color steganalysis; modern security benchmarking.
DIV2K [77]	High-Resolution (2K)	1000	High-fidelity watermarking; super-resolution.
CelebA-HQ [79]	Human Faces	30 k	Deepfake detection; identity protection; generative tasks.

## 3. Deep Learning Data Hiding Architectures

**Figure 4:**
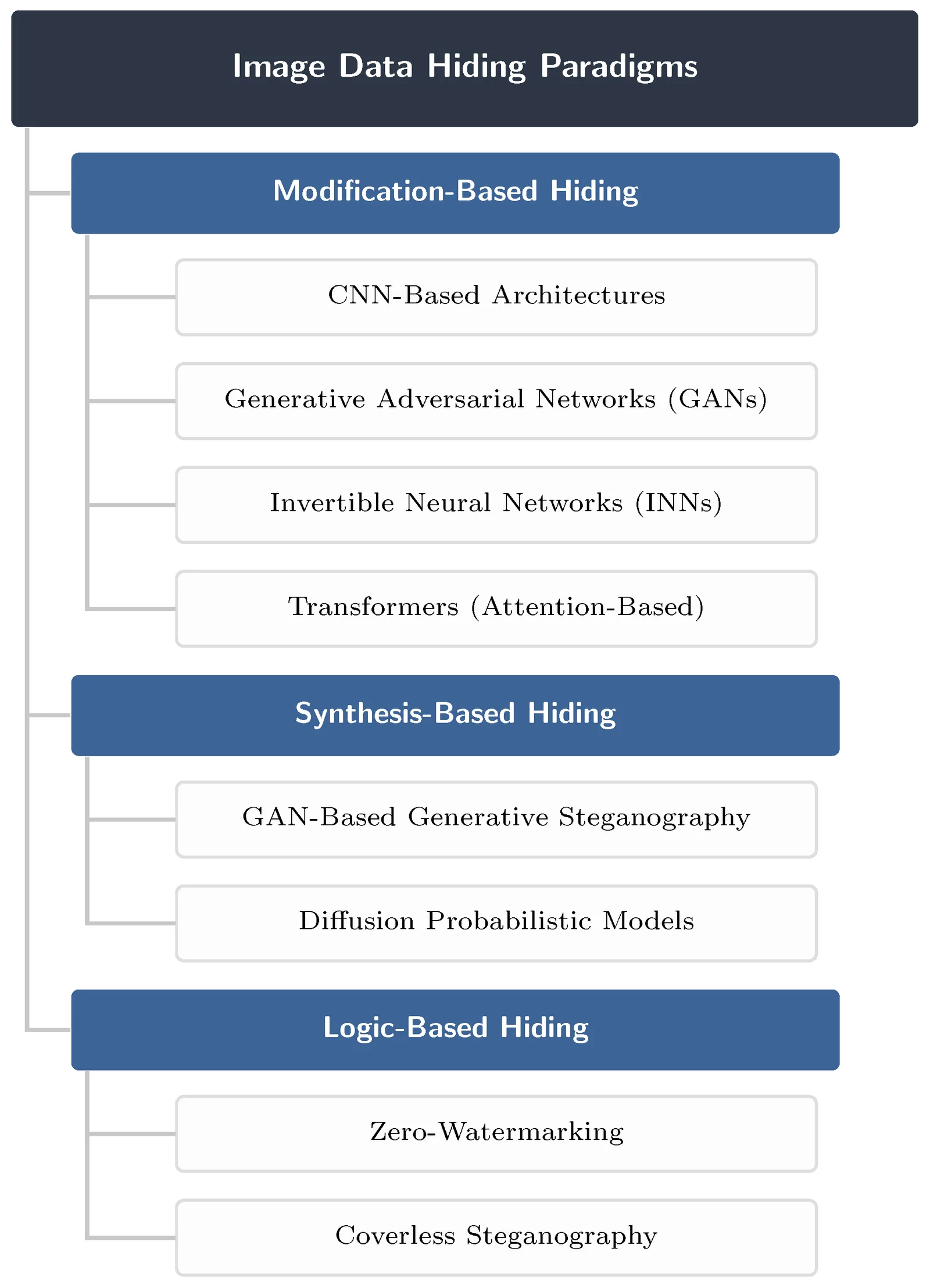
Proposed hierarchical taxonomy of image data hiding approaches.

This section presents a systematic review of deep learning-based image data hiding, organized according to the three-paradigm operational taxonomy introduced in Section 1: modification-based, synthesis-based, and logic-based approaches (Figure 4).

Throughout this section, we primarily analyze methods qualitatively to identify their structural mechanisms, inherent trade-offs, and fundamental architectural limitations. This focus reflects an established benchmarking challenge in the literature: methods are often evaluated across disparate datasets, input resolutions, attack configurations, and JPEG quality factors, meaning that higher reported PSNR or bit accuracy frequently stems from lower payload capacities or weaker attack simulations rather than architectural superiority alone. Nevertheless, to provide empirical context and document the operational characteristics of modern data hiding, we consolidate the reported performance metrics, independent third-party evaluations, and computational profiles of 16 representative works.

Importantly, the operational trade-offs and vulnerabilities identified throughout this section are governed primarily by class-wide architectural constraints rather than isolated implementation choices. For instance, sensitivity to lossy compression in spatial CNNs or the substantial computational footprint of iterative diffusion models represents inherent structural boundaries shared across their respective families. Accordingly, while individual framework contributions and specific constraints are cataloged in our milestone tables (Table 4, Table 5 and Table 6), our narrative focuses on analyzing how each overarching paradigm navigates the core trade-off triangle (Figure 3) across capacity, imperceptibility, and robustness.

To provide a consistent organization, we classify the reviewed literature using a two-tier framework. The primary tier classifies paradigms based on how the carrier image is handled during embedding, while the secondary tier organizes methods internally based on their architectural development and functional objectives.

This primary classification depends strictly on the carrier’s role during embedding, independent of the underlying network architecture:

- Modification-Based: The payload is embedded by directly modifying the pixels or transform-domain coefficients of a pre-existing cover image.
- Synthesis-Based: The stego image is synthesized as part of the embedding process (e.g., from noise or a latent trajectory) rather than through editing. This includes image-to-image (I2I) diffusion methods; although they accept a source image as conditioning, the final stego image is produced via iterative generative denoising rather than direct pixel-level modification.
- Logic-Based: The carrier is neither modified nor generated. This encompasses zero-watermarking (mathematically deriving a verification key from deep features, leaving the original image untouched) and coverless steganography (retrieving an unmodified database image whose intrinsic features map to the payload).

Within each primary paradigm, methods are organized into three developmental stages:

- Foundational Pipelines: Baseline formulations establishing the core embedding and extraction framework.
- Functional Optimizations and Hybrid Architectures: Algorithmic and structural mechanisms (such as attention, adversarial training, or optimized losses) designed to improve trade-offs among capacity, imperceptibility, and robustness. This includes hybrid models (e.g., CNN–Transformer and INN–Transformer fusions), which are categorized under the later architectural component in the evolutionary sequence when that component represents the primary novelty resolving a predecessor’s bottleneck.
- Specialized and Domain-Specific Variants: Adaptations of established architectures to specific data modalities (medical, RAW, or geospatial imagery) or deployment environments (mobile, real-time, or resource-constrained hardware).

This structured progression allows the survey to transition logically from fundamental embedding mechanisms, through functional and hybrid developments, to specialized real-world application scenarios.

### 3.1. Modification-Based Hiding

Modification-based methods embed a payload by altering the pixels or transform-domain coefficients of an existing cover image. This paradigm predates deep learning and has traditionally relied on explicitly designed embedding rules, distortion functions, and optimization procedures. In classical adaptive steganography, for example, each potential modification is associated with an embedding cost that estimates its impact on perceptual quality or statistical detectability. Lower-cost regions are preferentially selected for embedding, allowing the modification pattern to adapt to image content. Transform-domain watermarking follows a related principle by selecting coefficients or frequency bands according to their expected perceptual and robustness characteristics. These approaches provide well-defined behavior, low computational complexity, and relatively straightforward deployment, but their performance is ultimately constrained by the assumptions encoded in the manually designed embedding model.

A fundamental development introduced by deep learning is the possibility of learning the embedding strategy from data rather than specifying it explicitly. Neural networks can learn content-dependent modification patterns jointly with the payload representation and extraction process. In this context, embedding cost can be either explicitly learned as a spatial or transform-domain cost map, or implicitly represented by the learned embedding transformation and its optimization objective. This allows the embedding strategy to adapt to image structures that are difficult to characterize using fixed analytical models. Furthermore, perceptual fidelity, payload recovery, and robustness can be optimized jointly during training, enabling the embedding strategy to be tailored to the target operating conditions and attack models.

This transition does not eliminate the fundamental trade-offs of modification-based hiding, but changes how they are addressed. Increasing capacity can still increase embedding distortion, while stronger robustness can require a larger or more structured modification signal. Deep learning provides a more flexible mechanism for navigating these competing objectives by learning their relationship from training data. It can therefore improve adaptivity and enable joint optimization of properties that traditionally required separate design choices. However, this flexibility comes at the cost of increased training requirements, computational complexity, and reduced interpretability. The learned embedding strategy is also dependent on the training distribution, payload configuration, and distortions represented during optimization, which can limit generalization to unseen image content or attack conditions. Consequently, deep learning-based modification methods trade the explicit, interpretable, and computationally efficient nature of traditional embedding costs for a data-driven embedding strategy with greater flexibility but higher training and deployment complexity.

The evolution of modification-based hiding can therefore be viewed as a progression from manually designed embedding costs and representations toward increasingly data-driven embedding strategies. Early deep learning approaches primarily learned the embedding and extraction transformations jointly, while subsequent methods introduced attention mechanisms, adversarial objectives, invertible architectures, transformers, and other mechanisms to address specific limitations in capacity, imperceptibility, robustness, and computational efficiency. The following subsections organize these developments according to their architectural progression and the particular limitations they aim to address.

#### 3.1.1. Convolutional Neural Networks

The shift from traditional heuristics to deep learning in image data hiding marks a fundamental methodological evolution. Rather than manually designing embedding rules, deep learning systems learn to encode information within the optimal high-dimensional feature space of the cover medium. This progression has been motivated by the need to overcome key architectural limitations present in earlier approaches.

CNNs marked the first major transition toward fully end-to-end learnable data hiding. By leveraging the hierarchical feature extraction capabilities of convolutions, these networks jointly optimize the encoding of secret information into cover images, thereby substituting fixed algorithms with differentiable optimization. Early research framed this task as an image-to-image translation problem, typically utilizing autoencoder architectures to map cover images and payloads onto stego images.

Encoder-Decoder Framework

The foundational architecture for CNN-based hiding follows a three-stage pipeline: a preparation network transforms the secret into embeddable features, a hiding network fuses these features with the cover, and a reveal network extracts the payload. Kandi et al. [83] pioneered CNN-based autoencoders for image-in-image hiding, though their method remains non-blind, requiring the original cover for detection. Baluja [84] eliminated this constraint, proposing the first blind framework capable of concealing full-size RGB images without cover dependency during decoding. Although it achieved high visual fidelity, Baluja’s model lacked explicit robustness training against channel distortions.

Subsequent research systematically addressed this limitation. Mun et al. [85] proposed a blind CNN-based watermarking method for embedding and extracting binary message watermarks. Extending this, Ahmadi et al. [86] introduced ReDMark, a residual diffusion framework in which the watermark is embedded into the residual stream of a fully convolutional network, ensuring it is distributed across the entire spatial domain rather than concentrated in localized regions. Circular convolutional layers further enforce uniform diffusion by wrapping the kernel periodically across image boundaries, eliminating the edge artifacts introduced by standard zero-padded convolutions.

To train for robustness explicitly, Lee et al. [87] integrated a noise simulation layer between the encoder and decoder during training. This forced the network to learn redundant features robust to specific distortions (e.g., JPEG, cropping). However, simulated JPEG remains a differentiable approximation of the real operation. Jia et al. [88] addressed this gap with Mini-Batch of Real and Simulated JPEG compression (MBRS), randomly alternating the noise layer between real JPEG, simulated JPEG, and a noise-free identity layer across different mini-batches, thus directly exposing the decoder to real non-differential JPEG compression while maintaining end-to-end trainability, achieving near-zero BER under JPEG at Quality Factor (QF) 50.

Extending encoder–decoder principles to the recovery stage, Singh and Singh [89] and Xi et al. [90] integrated denoising autoencoders and super-resolution modules directly into the extraction pipeline. Zhao et al. [91] further advanced this concept with CAML, a context-aware multitask framework that jointly learns mask estimation and inpainting. By coupling these tasks, CAML enhances both the localization of embedding regions and the restoration of the cover image. Finally, expanding functional capacity, Sood et al. [92] developed a dual-watermarking framework. Leveraging a component-based design, their architecture utilizes parallel preparation networks to conceal two distinct secret images within a single cover, secured by chaotic encryption (3D Logistic and Chirikov maps).

Attention-Guided Embedding

Rather than embedding uniformly across the spatial domain, attention mechanisms enable CNNs to identify perceptually optimal regions where modifications are naturally masked by texture complexity. Yin et al. [93] introduced the Bidirectional-Interactive and Context-Aware (BICA) network, employing attention to target high-frequency textures where the Human Visual System exhibits reduced sensitivity. This approach directly addresses the imperceptibility-capacity trade-off by concentrating payload bits in regions where distortion is least perceptible, rather than distributing them uniformly where smooth areas may introduce visible artifacts.

Guidance-Based Hybrid Approaches

Beyond acting as end-to-end generators, CNNs are also employed to enhance traditional algorithms by identifying optimal embedding regions. This guidance-based approach leverages the semantic understanding of CNNs while relying on established embedding rules. Meng et al. [94] utilized Faster R-CNN [95] with a Region Proposal Network (RPN) to detect complex, texture-rich regions suitable for spatial-domain algorithms like HUGO [96], WOW [97], and S-UNIWARD [98]. By targeting these regions, the method avoids smooth areas where noise artifacts are easily perceptible.

Deep feature extraction has also been adapted to reversible data hiding schemes. Eltoukhy et al. [99] proposed RDHNet, a hybrid framework that utilizes a pre-trained AlexNet [100] combined with the Watershed Transform to generate a topological feature map of the cover image. This deep feature representation guides a histogram-based embedding strategy, allowing for high-capacity payload insertion secured by L-shaped Fractal Tromino encryption, while ensuring the original cover can be losslessly reconstructed.

Domain-Specific Extensions

The encoder–decoder framework has been adapted to specialized input domains that impose distinct physical or regulatory constraints on the watermarking process. Extending this pipeline to the sensor level, Hu et al. [101] addressed RAW image watermarking with DRAW, utilizing multi-frequency feature fusion and hybrid attack simulation to ensure watermarks survive the non-linear transformations of Image Signal Processing (ISP) pipelines and enable tamper localization. Complementing this, Fu et al. [102] proposed RAWIW, integrating the watermarking module directly with a differentiable ISP simulation model, guaranteeing that the copyright signal remains detectable in the final rendered RGB output.

In the geospatial domain, Wang et al. [103] addressed the challenges of large-scale satellite imagery via a dual-stage training framework incorporating DWT-based frequency embedding and residual attention, enabling the model to learn scale-invariant features necessary for protecting high-resolution remote sensing datasets. Addressing specialized content formats, Yang et al. [104] introduced a segmentation-based synchronization scheme for alpha-matte images common in mobile editing pipelines, designed to resist the irregular cropping and compositing operations prevalent on social media platforms.

Summary and Analysis of CNN Architectures

CNN-based methods established the foundation for end-to-end differentiable optimization in image data hiding, replacing hand-crafted embedding rules with learned feature representations. However, these architectures are constrained by three fundamental limitations.

First, non-bijective mapping and information loss: Standard CNNs employ downsampling operations (pooling, strided convolutions) and non-linear activations (ReLU) that are mathematically non-invertible, forcing decoders to approximate rather than analytically recover the payload, a fundamental constraint that limits extraction accuracy under channel distortion.

Second, restricted receptive field: CNNs process images through sliding windows with limited spatial context, making them vulnerable to large-scale geometric transformations (rotation, scaling) where local feature patterns become spatially desynchronized.

Finally, capacity-fidelity conflict: Increasing payload density in spatial CNNs introduces visible high-frequency artifacts or color shifts, as pixel-wise reconstruction losses (MSE, L1) penalize perceptual distortions equally across all frequencies. This motivates the shift toward Adversarial Training, where discriminators enforce perceptual rather than pixel-level similarity, allowing higher capacity without visible degradation.

#### 3.1.2. Adversarial Models

To overcome the blurring artifacts and low-frequency bias caused by pixel-wise MSE losses in standard CNNs, the field transitioned to Generative Adversarial Networks (GANs), which introduce an adversarial discriminator to optimize for statistical and perceptual imperceptibility. Rather than minimizing pixel-wise reconstruction error, the adversarial framework trains a generator to produce stego images that a discriminator cannot distinguish from natural covers, thus shifting the objective from numerical fidelity toward statistical imperceptibility. Critically, the generator in these frameworks still operates on an existing cover image, modifying its pixel values to embed the payload. The adversarial loss supplements, rather than replaces, the embedding mechanism, addressing the low-frequency bias and blurring artifacts that result from pixel-wise losses such as MSE, which penalize all frequency components equally and consequently favor smooth, averaged reconstructions.

Foundational Adversarial Frameworks

The pivotal work in this domain is HiDDeN by Zhu et al. [23], which established the standard encoder–decoder–discriminator architecture. HiDDeN was the first to jointly optimize imperceptibility and robustness by inserting a noise layer between the encoder and decoder. Crucially, this noise layer is differentiable, allowing gradients to backpropagate from the decoder to the encoder, thereby forcing the network to learn redundant features robust to specific distortions during training. Although it achieved blind decoding, HiDDeN suffered from low capacity (∼0.2 bpp) and limited generalization. Despite these constraints, it serves as the foundation for numerous successors [105,106,107]. Addressing the capacity bottleneck, Zhang et al. [108] introduced SteganoGAN, which refined the loss functions and payload handling to allow for denser embedding without inducing the generator collapse common in earlier iterations.

Parallel to HiDDeN, Hayes and Danezis [109] approached the problem from a purely security-oriented perspective, introducing a three-party adversarial framework in which Alice (encoder), Bob (decoder), and Eve (steganalyzer) are trained simultaneously. Rather than optimizing for perceptual quality, the objective is statistical undetectability: Alice is penalized directly by Eve’s classification performance, forcing the embedding to remain indistinguishable from the natural image distribution.

Optimization and Robustness Enhancement

Subsequent research focused on stabilizing training and improving robustness against complex distortions. To enhance robustness, Wen and Aydore [110] introduced ROMark, replacing standard training with a min-max robust optimization approach. By focusing on minimizing decoding loss under worst-case distortions, ROMark significantly outperforms the average-case optimization of HiDDeN.

Addressing the problem of multi-scale robustness, Bui et al. [111] proposed TrustMark. This framework embeds a residual watermark at a standardized resolution and then scales it back to the original image size, ensuring that the watermark remains detectable regardless of the image’s final display resolution. Hybrid optimization strategies have also emerged. Martin et al. [112] integrated genetic algorithms with GANs to evolve the generator architecture itself, automatically finding the optimal network structure to minimize visual alteration for specific cover images.

Attention-Guided Regional Embedding

To improve both imperceptibility and security, modern GAN-based frameworks increasingly leverage attention mechanisms to identify optimal embedding regions, targeting texture-rich areas where modifications are less perceptible. Yu et al. [113] established this trend by integrating a ResNet-based attention module to guide embedding, whereas Huang et al. [106] proposed a Feature Fusion Module combined with attention masks. Recently, Liang et al. [114] pushed this concept further with UMSA-GAN, utilizing Residual Channel-Spatial Attention (Res-CBAM) to target high-frequency textures for improved steganalysis resistance.

Expanding this attention hierarchy to the recovery process, Luo et al. [115] introduced RME-GAN, which utilizes Probability Spatial Attention (PSAM) for adaptive region selection while incorporating Multidimensional Attention (MAM) into the extractor to suppress transmission noise features. Building on this, Yan et al. [116] integrated Dynamic Swin Attention into an adversarial framework to improve concealment against deep steganalyzers. Their architecture handles global noise and local artifacts in distinct stages, utilizing differential features at the decoder to compensate for information loss.

Attention principles have also been applied to reversible steganography. For instance, Xie et al. [117] employ Grad-CAM to identify significant regions that a specialized restorer module uses to ensure perfect cover recovery.

Security Extensions

Beyond perceptual quality optimization, several works integrate cryptographic security and reversible recovery capabilities into adversarial frameworks. Singh et al. [118] proposed a dual-layer security framework using chaotic maps and Random SVD to provide simultaneous copyright protection and tamper localization. Similarly, Gao et al. [119] combined SE-ResNet attention with secret sharing and Reed-Solomon ECC. By utilizing chaotic scrambling to disperse localized distortions, this hybrid framework achieves perfect secret recovery even under a 25% region crop attack demonstrating that cryptographic redundancy can compensate for adversarial training’s inherent fragility.

Deployment-Specific Extensions

For resource-constrained and domain-specific deployment scenarios, several frameworks adapt adversarial training to computational and regulatory constraints. Singh et al. [120] introduced a split-learning GAN framework that offloads heavy adversarial processing from edge devices, maintaining watermark robustness under the strict computational constraints of IoT networks. Extending adversarial frameworks to medical IoT, He et al. [121] developed DAS-GAN, a dual-adversarial framework for grayscale medical images that balances adversarial properties across both spatial and transform domains.

Summary and Analysis of Adversarial Methods

The integration of adversarial training marked a pivotal shift from pixel-wise error minimization to perceptual optimization, effectively solving the “blurring” artifacts common in early CNNs. However, this paradigm introduces three fundamental limitations.

First, convergence instability: The min-max game between the generator and discriminator is notoriously difficult to optimize, often leading to mode collapse where the generator collapses to a low-diversity output distribution, repeatedly producing similar samples regardless of input variation.

Second, statistical detectability: Whereas GAN-generated steganography is visually superior, the upsampling layers in the generator often leave distinct spectral artifacts that are easily detected by modern deep steganalysis tools like SRNet [68].

Finally, standard GAN generators are non-invertible functions. They inherently destroy the original pixel information during the forward pass, making the mathematically lossless recovery of the cover impossible. This lack of reversibility prevents their use in medical or forensic applications, necessitating the shift toward INNs, which offer a mathematically rigorous solution to this problem.

#### 3.1.3. Invertible Neural Networks

Because both CNNs and GANs employ mathematically non-invertible operations that permanently destroy high-frequency cover details, INNs emerged to enable bijective, near-lossless data hiding. Unlike traditional networks, INNs learn a bijective mapping $f:R^{d}\to R^{d}$, allowing both embedding (forward pass) and extraction (backward pass) to be performed within a single unified model.

This architectural bijectivity provides a theoretical basis for reversible embedding and extraction; however, it should not be interpreted as guaranteeing pixel-wise lossless recovery in practical image representations. In particular, three notions of reversibility should be distinguished. At the architectural level, an INN can implement a mathematically bijective mapping, meaning that the continuous-valued network representation can, in principle, be exactly recovered through the inverse transformation. At the image-reconstruction level, however, the recovered image is typically only near-lossless because the network operates using finite-precision floating-point arithmetic and the resulting output must ultimately be represented as a discrete image, commonly using 8-bit integer values. The conversion between continuous floating-point representations and discrete integer-valued pixels can introduce rounding and quantization errors, even when the underlying network is theoretically invertible. Therefore, claims of lossless recovery in conventional floating-point INNs should be understood primarily in the architectural or theoretical sense unless exact recovery in the discrete image domain is explicitly demonstrated.

This distinction is particularly important for reversible image hiding, where the objective is not merely to reconstruct the secret image accurately but also to recover the original cover image exactly. Integer-based invertible architectures, such as CRMark [122], address this implementation-level limitation by performing the invertible transformation directly in the discrete integer domain, thereby avoiding the rounding errors introduced by floating-point-to-integer conversion. Such approaches provide a stronger form of reversibility and are therefore discussed separately when considering exact pixel-wise recovery.

High-Capacity Reversible Steganography

The primary advantage of INNs is their bijective mapping, implemented via affine coupling layers [123], which guarantees theoretically lossless recovery in distortion-free channels (ignoring quantization error and lost information). This enables both lossless cover recovery and analytically exact payload extraction, a property that standard CNNs and GANs cannot achieve due to their non-invertible downsampling operations. The pioneering HiNet [124] introduced INNs for reversible image-in-image hiding. Images are first decomposed using DWT into low- and high-frequency sub-bands, which are then processed through shared invertible concealing blocks. By utilizing the same weights for hiding and revealing, HiNet achieved high-fidelity reconstruction for both the cover and secret. A low-frequency loss is applied, ensuring that the stego image’s low-frequency components remain visually similar to the original, improving imperceptibility.

Scaling this framework further, Lu et al. [125] designed a framework capable of concealing up to five full-resolution images inside a single cover while maintaining a PSNR above 30 dB. By increasing the number of hidden image channels, their model effectively showcased the scalability of INNs for multi-secret embedding, achieving higher hiding capacity without compromising image quality. Extending multi-image hiding toward practical robustness, Guan et al. [126] proposed DeepMIH, which introduced a learnable bijective mapping for hiding multiple images while modeling the distribution of lost high-frequency information *r* as a fixed Gaussian prior. Wang et al. [127] further adapted INNs for lossless steganography by formulating the problem as a super-resolution task. Their model utilizes a flow-based projection module to map secret images into a latent distribution, allowing for the embedding of high-resolution secrets into lower-resolution covers with mathematically perfect extraction.

Robustness and Differentiable Attack Simulation

Whereas early INNs focused on capacity, subsequent research addressed their fragility to channel distortions. Unlike image-in-image hiding, bit-level watermarking requires embedding discrete binary sequences rather than continuous pixel values. To adapt INNs for this task, Xu et al. [128] introduced a message normalization layer that maps binary bitstreams into image-shaped tensors compatible with invertible blocks.

A critical challenge in robust watermarking is optimizing against non-differentiable attacks like JPEG compression. Ye et al. [129] addressed this in DBMark. To bypass the gradient blockage caused by JPEG, DBMark employs a forward Attack Simulation Layer (ASL) inspired by [130]. During the forward pass, the ASL applies real distortions (pseudo-noise) to the encoded image, and during backpropagation, the ASL is skipped, allowing the network to approximate gradients and learn robustness against quantization and compression without breaking the training loop.

Defense Against AIGC and Generative Editing

The rise in AIGC editing tools (e.g., inpainting, style transfer) poses a new threat to watermark survival. Zhang et al. [131] introduced EditGuard to counter this, proposing a dual-watermarking scheme: a semi-fragile watermark for tamper localization and a robust watermark for copyright protection. To solve the lost information problem in INNs, EditGuard utilizes a prompt-conditioned latent generator to predict the posterior distribution of the vanished information $\hat{z}$. Unlike standard methods [125,126] that assume a fixed Gaussian distribution, EditGuard employs learnable degradation prompts in the form of embedding tensors that represent various distortion types. By calculating dynamic weights based on the features of the received image, the model adaptively integrates these prompts to enrich the reconstruction context, ensuring the watermark can be recovered even after complex semantic or channel-based degradations. Complementing this direction, Huo et al. [132] proposed a fitting model that generates an auxiliary variable *z* during extraction to approximate the lost high-frequency residual, directly addressing the Gaussian prior assumption inherited from HiNet and DeepMIH.

This concept was further evolved in OmniGuard [133]. Unlike EditGuard’s fixed localization watermark, OmniGuard introduces an adaptive watermark transform that embeds localized information in a content-aware manner, decoupling the encoding and decoding ends via a passive blind extractor. Furthermore, it incorporates a lightweight AIGC-editing simulation layer to enhance robustness against both global editing and local inpainting attacks, significantly improving fidelity and copyright extraction accuracy over EditGuard.

Hybrid and Specialized Architectures

A fundamental limitation of standard flow models is the quantization error: INNs operate on floating-point numbers, but images are stored as 8-bit integers. This quantization destroys perfect reversibility. To address this, Chen et al. [122] proposed CRMark, which utilizes Integer Discrete Flows [134] to prevent rounding errors. CRMark employs a two-stage strategy: an integer discrete invertible network embeds the robust watermark, while a secondary reversible step embeds auxiliary information (overflow maps) to guarantee mathematically lossless cover recovery in lossless channels.

Recent works have extended INN principles to hybrid designs, enhancing both security and imperceptibility. Huo et al. [135] introduced Fully Reversible Image Hiding (FRIH). Unlike standard INNs that swap cover frequencies for payload, FRIH generates a minimal-norm disturbance tensor applied via a subtractive operation, preserving more of the original cover structure while maintaining invertibility. By reconstructing input dimensionality and minimizing tensor coupling, the method ensures lossless recovery and strong resistance to steganalysis.

Shi et al. [136] introduced StarINN, which integrates the Star operation [137] to enhance feature representation and accelerate convergence. The framework uses separate subnetworks for the secret and cover images, where the secret branch employs stacked StarBlocks for efficient high-dimensional mapping, and the cover branch combines DenseBlocks [138] and NAFBlocks [139] for stable feature extraction. This design improves embedding capacity, visual quality, and recovery accuracy compared to previous INN-based approaches. Similarly, Wang et al. [140] demonstrated the utility of combining architectures by proposing a hybrid invertible–GAN framework. By utilizing multiple extraction designs, their method significantly improves extraction reliability and robustness against steganalysis compared to pure INN baselines.

For depth-image-based rendering (DIBR), Chen et al. [141] proposed MCANet, which employs cross-modal attention to align depth maps with central views within an invertible framework, ensuring the watermark survives 3D view synthesis while maintaining lossless cover recovery.

Together, these hybrid and domain-specific designs illustrate that the INN framework is not a fixed architecture but an adaptable mathematical backbone applicable across diverse media types and embedding scenarios.

Summary and Analysis of Invertible Methods

The adoption of INNs revolutionized data hiding by offering a mathematically rigorous solution to the capacity-fidelity trade-off, enabling massive payload embedding with near-perfect reconstruction. However, despite their dominance in payload density, this architecture suffers from two intrinsic limitations.

First, the lost information *r* dilemma: to hide data, INNs effectively swap out high-frequency cover details *r* for the payload. Recovering the cover requires estimating this distribution, typically assuming Gaussianity. However, when the stego image undergoes complex distortions like AIGC editing, this assumption breaks, leading to significant artifacts in the recovered cover.

Second, quantization error: the discrepancy between floating-point network operations and integer image formats introduces rounding noise. This noise accumulates in deep flows and degrades recoverability unless specialized Integer Discrete Flows are employed [122].

Despite their architectural differences, CNNs, GANs, and INNs all rely on local feature extraction through convolutional operations, either as standalone encoders (CNNs, GANs) or within invertible coupling functions (INNs). This shared dependency on sliding-window convolutions restricts their receptive fields, making them susceptible to geometric attacks where spatial alignment is disrupted. Transformer architectures address this constraint through self-attention mechanisms that capture dependencies across the full image plane, enabling robust watermark embedding under geometric transformations.

#### 3.1.4. Transformers

While INNs resolved capacity and reversibility, CNNs, GANs, and INNs all share a reliance on sliding-window convolutions with localized receptive fields, rendering them fragile to geometric desynchronization (cropping, rotation); this limitation drove the adoption of Vision Transformers (ViTs) [142] for global spatial synchronization. Transformer architectures were originally developed to model long-range dependencies in natural language processing (NLP) tasks [143]. Their subsequent adaptation to the visual domain, specifically through the ViTs, has recently gained considerable attention in image data hiding. This adaptation addresses a fundamental limitation shared by CNNs, GANs, and INNs: whereas these architectures differ in their optimization objectives, they all rely on convolutional feature extractors with restricted local receptive fields. By leveraging self-attention mechanisms across the entire image, Transformers enable embedding into globally correlated features, providing robustness against geometric attacks (e.g., cropping, rotation, scaling) where local feature alignment is destroyed.

Pure Vision Transformers Architectures

The ViT architecture processes images as sequences of patches, enabling self-attention mechanisms to capture global dependencies. This has inspired a new class of embedding frameworks that prioritize global semantic coherence over local texture optimization. Liu et al. [144] leveraged this property to design a screen-shooting-resistant watermarking scheme. By utilizing multi-head cross-attention, their model effectively synchronizes the watermark signal across the entire image plane, integrating it with a U-Net decoder to mitigate information loss. Although it demonstrated robustness under various conditions, watermark embedding was concentrated near image edges, reducing recovery accuracy for partially captured images (cropping).

Beyond general robustness, Transformers are redefining security in sensitive domains. Nanammal et al. [145] introduced ViT-U-Net, a framework for medical image integrity. Unlike standard global embedding, ViT-U-Net employs Adaptive Hierarchical Spatial Attention to distinctively separate Region of Interest (ROI) diagnostic areas from the background, embedding data only in non-diagnostic regions. This demonstrates how attention masks can enforce strict, semantically aware security policies that CNNs struggle to learn without explicit supervision.

Channel and Spatial Variants

Although pure ViTs apply self-attention across spatial positions, recent works have extended attention mechanisms to the channel dimension and spatial hierarchies to refine feature selection. Zhou et al. [146] introduced CAISFormer, shifting the focus from spatial to channel-wise attention. By incorporating a Channel Self-Attention Module (CSAM), the network reweights feature channels based on their importance, whereas a Global–Local Aggregation Module (GLAM) directs embedding toward texture-rich regions. This design allows the network to hide larger payloads by exploiting inter-channel correlations that spatial CNNs often overlook.

Generative Transformer Frameworks

Extending Transformers beyond feature extraction, several works integrate adversarial training and conditional generation to enhance both imperceptibility and security. Xiao et al. [147] integrated adversarial learning with pure attention mechanisms, replacing standard CNNs with a transformer-based adversarial network. Their framework utilizes a WGAN discriminator to stabilize training while simulating real-world noise and geometric distortions to ensure robustness.

Expanding the generative capabilities of Transformers further, Zhang et al. [148] proposed IHST, framing data hiding as a conditional image reprogramming task. By employing encoding, transfer, and synthesis modules, IHST stylizes the latent representations of the cover to embed a secret image of equal resolution. This approach leverages the global modeling capacity of transformers to fuse latent features across the full image plane without patch-boundary discontinuities, maximizing payload capacity while maintaining resistance to statistical detection.

Hybrid CNN-Transformer Architectures

To mitigate the high computational complexity of pure transformers and address their limitation in capturing fine-grained local features, a hybrid trend has emerged, combining CNNs (for local detail) with Swin Transformers (for efficient global context). Whereas pure ViT attention scales quadratically ($O(N^{2})$), the Swin Transformer variant [149] mitigates this via shifted window attention to linear complexity (O(N)), motivating its adoption in hybrid designs.

Aberna and Agilandeeswari [41] applied Swin Transformers alongside the Quaternion Dual-Tree Complex Wavelet Transform (QDT-CWT) for HDR watermarking. Here, the Transformer acts as a robust feature extractor, while the quaternion transform helps identify optimal embedding regions in high-dynamic-range content. Wan et al. [40] extended this quaternion-Transformer direction to macrophotography, developing a quaternion-based attention mechanism that guides embedding into perceptually robust regions while avoiding disruption of the shallow depth of field characteristic of macro imagery.

Addressing the computational constraints of these architectures, Younis et al. [150] proposed AttenHideNet, a lightweight hybrid U-Net employing soft attention mechanisms and YUV color space embedding. By distributing the secret across all three YUV channels simultaneously, the framework achieves image-in-image steganographic capacity of 24 bpp (equivalent to concealing a full RGB image), while maintaining low inference latency of 18 ms per image, making it suitable for resource-constrained deployment.

Segmentation-Based Localized Watermarking

A notable architectural departure from global extraction reframes watermarking as a dense prediction task. Standard encoder–decoder extractors produce a single global payload decision, which degrades whenever unwatermarked pixels dilute the embedded signal—a fundamental vulnerability to cropping, splicing, and generative inpainting. Sander et al. [151] addressed this directly with the Watermark Anything Model (WAM), which pairs a ViT-Base encoder with a convolutional pixel decoder to produce a per-pixel detection score and message vector. This design enables the localization of watermarked regions regardless of what fraction of the image they occupy. By applying DBSCAN clustering to the pixel-level outputs, WAM further enables the simultaneous extraction of multiple distinct payloads from different regions of a single composite image, a capability not previously demonstrated in image watermarking. Empirically, WAM achieves near-perfect True Positive Rates (TPR) under aggressive inpainting and splicing attacks.

INN-Transformer Integration

Bridging two major architectural paradigms, He et al. [152] introduced IWFormer, which merges INNs with a custom Affine Transformer. Unlike standard hybrids that simply concatenate CNN and Transformer features, IWFormer replaces the DenseNet blocks inside the INN’s affine coupling functions with self-attention mechanisms. This integration ensures tight coupling of embedding and extraction via the INN’s mathematical invertibility, whereas the Transformer captures global correlations between cover and watermark features. The result is a framework that combines INN’s lossless recoverability with Transformer’s geometric robustness, guiding embedding toward inconspicuous regions while maximizing both imperceptibility and recovery accuracy.

Summary and Analysis of Transformer Methods

Transformer-based architectures represent a significant evolution in modification-based data hiding, utilizing self-attention mechanisms to capture long-range dependencies that convolutional networks miss. By processing images as patch sequences rather than through sliding windows, Transformers enable watermark synchronization across the full spatial domain, providing robustness against geometric attacks where local feature alignment is destroyed. However, their adoption introduces three fundamental trade-offs.

First, computational complexity: Transformer-based methods carry a substantially higher computational footprint than CNN- or INN-based approaches. Even efficient variants such as Swin Transformers with windowed attention, and hybrid CNN-Transformer architectures, introduce considerably greater training and inference costs compared to single-pass convolutional encoders, which limits their practical applicability in resource-constrained or real-time deployment scenarios.

Second, inductive bias and data efficiency: Unlike CNNs, which possess inherent inductive biases for translation invariance, Transformers require significantly larger datasets to generalize effectively. Without massive-scale pre-training, they are prone to overfitting on smaller watermarking datasets.

Finally, local detail preservation: Although superior at modeling global structure through self-attention, patch-based ViTs can struggle with preserving high-frequency edges and fine textures compared to the sliding-window operations of CNNs. Patch tokenization inherently discards sub-patch spatial relationships, making it difficult to embed watermarks into localized high-frequency regions without introducing visible artifacts. Consequently, current research is converging toward hybrid architectures, which combine the efficiency of CNNs for local texture extraction with the robustness of Swin Transformers for global synchronization, as demonstrated by AttenHideNet and IWFormer.

#### 3.1.5. Summary and Analysis of Modification-Based Methods

Modification-based data hiding has evolved through four distinct architectural paradigms, each addressing specific limitations of its predecessor yet introducing new trade-offs. Table 4 summarizes the milestone frameworks across CNNs, GANs, INNs, and Transformers, highlighting their core mechanisms and inherent constraints.

CNNs established the foundation for end-to-end differentiable optimization, enabling learned feature representations to replace hand-crafted embedding rules. However, their non-invertible downsampling operations and local receptive fields fundamentally limited both recoverability and geometric robustness. GANs addressed the perceptual quality problem by shifting from pixel-wise reconstruction losses to adversarial training, achieving statistical imperceptibility at the cost of training instability and spectral detectability. INNs resolved the recoverability constraint through bijective mapping, enabling near-lossless cover recovery and massive payload capacity, yet remained vulnerable to the Gaussian prior assumption and quantization error. Finally, ViTs introduced global self-attention to overcome the local receptive field bottleneck, providing robustness against geometric desynchronization. Whereas pure ViTs incur quadratic computational complexity, current research has shifted toward efficient variants (Swin Transformers with windowed attention) and hybrid architectures (CNN-Transformer fusion) that achieve global modeling at reduced computational cost.

Despite their architectural diversity, these paradigms share a common constraint: no single architecture can simultaneously maximize capacity, imperceptibility, and robustness while maintaining recoverability and computational efficiency. This trade-off structure is governed by information theory rather than engineering limitations. CNNs and GANs sacrifice recoverability for embedding flexibility; INNs sacrifice robustness for lossless recovery; pure ViTs sacrifice computational efficiency for global context. Current research increasingly recognizes this trilemma as fundamental, driving the shift toward hybrid frameworks that strategically combine complementary strengths. IWFormer merges INN invertibility with Transformer global attention; AttenHideNet fuses CNN local features with Swin windowed attention; CRMark integrates Integer Discrete Flows to bridge the floating-point-to-integer gap. These hybrid architectures represent the current frontier of modification-based data hiding, optimizing specific points within the capacity-imperceptibility-robustness trade-off space rather than attempting to escape it entirely.

**Table 4:** Milestone modification-based architectures in image data hiding (2017–2026). Type S (Steganography), W (Watermarking). Arch: CNN (ConvNet), GAN (Adversarial), INN (Invertible), ViT (Transformer).

Ref.	Year	Framework	Arch.	Type	Core Mechanism	Key Contribution	Main Limitation
Convolutional Foundations (CNN)							
[84]	2017	Baluja et al.	CNN	S	3-stage Autoencoder	Pioneer of blind full-size RGB-in-RGB hiding using deep features.	Extreme fragility to any channel noise or compression.
[86]	2020	ReDMark	CNN	W	Residual Diffusion	Embeds watermark into the residual stream for uniform spatial diffusion, reinforced by circular convolutions at image boundaries.	High embedding strength causes visible artifacts in smooth regions.
[91]	2025	CAML	CNN	W	Multi-task Inpainting	Joint mask estimation and embedding for localized tamper protection.	Robustness highly dependent on mask estimation accuracy.
Adversarial & Perceptual Optimization (GAN)							
[23]	2018	HiDDeN	GAN	W	Noise Simulation Layer	Established encoder–decoder–discriminator baseline with differentiable noise.	Fails under aggressive geometric desynchronization.
[108]	2019	SteganoGAN	GAN	S	Dense Connections	Scaled steganographic capacity via refined loss and dense feature reuse.	Poor resistance to lossy compression.
[111]	2023	TrustMark	GAN	W	Residual Scaling	Resolution-invariant watermark via standardized residual embedding.	Residual scaling may introduce artifacts at extreme resolution changes.
Reversible Paradigms (INN)							
[124]	2021	HiNet	INN	S	Wavelet Coupling	First theoretically lossless recovery of both cover and secret images.	Zero robustness; any channel distortion corrupts secret recovery.
[126]	2023	DeepMIH	INN	S	Bijective Multi-hiding	Learnable bijective mapping for multi-image hiding with Gaussian prior.	Gaussian prior assumption breaks under AIGC-based inpainting attacks.
[133]	2024	OmniGuard	INN	W	Adaptive Transform	Content-aware localization watermark with passive blind extractor.	Lightweight AIGC simulation may miss complex real-world editing chains.
Global Context & Attention Mechanisms (Transformer)							
[146]	2024	CAISFormer	ViT	S	Channel Self-Attention	Exploits inter-channel feature correlations for high-security embedding.	Susceptible to geometric shifts due to fixed patch-based partitioning.
[152]	2024	IWFormer	ViT	W	Affine Transformer	Merges INN invertibility with Transformer global context via Affine blocks.	Dual-architecture complexity increases training time and memory cost.
[144]	2025	ScreenShot	ViT	W	Cross-Attention	Multi-head cross-attention synchronizes watermark to resist Moiré distortion.	Recovery accuracy drops significantly for oblique capture angles.
[151]	2025	WAM	Hybrid	W	Dense Segmentation	Localized detection and multi-message extraction via pixel-level masking and DBSCAN clustering.	High extractor complexity (96M parameters) and limited payload capacity per region (32 bits).

### 3.2. Synthesis-Based Hiding

Unlike modification-based methods, synthesis-based approaches do not embed data into a pre-existing image. Instead, the carrier itself is generated as part of the hiding process, fundamentally reframing data hiding as a generative task rather than an editing one. This paradigm splits into two operationally distinct applications: generative steganography, where a new image is synthesized from scratch specifically to carry a payload, and proactive AIGC watermarking, where provenance indicators are embedded during the content creation process itself. Both are primarily enabled by DPMs, though early GAN-based generative methods established the conceptual foundation.

#### 3.2.1. GAN-Based Models

Before diffusion models established the current paradigm, GANs provided the first practical demonstrations of synthesis-based hiding. Volkhonskiy et al. [24] pioneered this direction by training a DCGAN to synthesize carrier images with statistical properties that are inherently more resistant to steganalysis, into which conventional embeddings are then applied. Rather than learning the embedding itself, the key insight is that GAN-generated images occupy a different region of the statistical distribution than natural photographs, causing steganalyzers trained on real images to fail on generated containers. Building on this, Meng et al. [25] proposed S-CycleGAN, which embeds messages during unpaired image-to-image translation, leveraging cycle consistency to preserve visual quality while resisting detection via adversarial loss. Expanding this to text-conditioned generation, Rehman et al. [153] demonstrated high-capacity hiding by generating anime-style images directly conditioned on secret text labels, achieving a reported capacity of 1088 bits.

Pushing GAN-based generative steganography toward provable security, Su et al. [154] proposed StegaStyleGAN, which introduces a secret data modulator that ensures the statistical distribution of generated stego images remains indistinguishable from that of natural images even when the test-time secret data distribution deviates from training. This provides a formal security guarantee absent from prior GAN-based methods, though pre-encoding and decoding steps introduce additional computational overhead at both the sender and receiver. These early GAN-based methods established the conceptual viability of generative steganography, though they remained constrained by mode collapse and training instability inherent to GAN optimization.

#### 3.2.2. Diffusion Probabilistic Models

A significant paradigm shift emerged in 2023–2024 with the adoption of DPMs [155] for data hiding. Unlike CNNs or GANs, which modify an existing image in a single pass, diffusion models generate images iteratively through a learned denoising process. This fundamentally reframes the data hiding task: data is not hidden behind pixels but embedded into the generative process itself.

Importantly, this architectural shift alters how methods interact with existing legacy assets (e.g., standard photography or medical scans). Existing images can participate in diffusion-based watermarking and steganographic pipelines through techniques such as deterministic diffusion inversion (e.g., DDIM), conditional generation, image inpainting, and latent-space optimization. However, applying DPMs to pre-existing images is constrained by distinct limitations. The stochastic nature of diffusion sampling can induce semantic drift or alter fine-grained details of a specific cover, requiring strict algorithmic controls (such as fixed initial noise seeds or frozen network backbones) to ensure reproducible outputs. Furthermore, the iterative denoising trajectory introduces a computational cost significantly higher than that of single-pass convolutional encoders.

Because of these constraints, while DPMs are applicable to existing-image protection, their unique architectural strengths have primarily driven the field toward two natively synthesis-based applications: generative steganography, where the cover image is synthesized from scratch specifically to carry a message, and proactive watermarking of AIGC, where provenance indicators are embedded during the content creation process itself.

Generative Steganography

Diffusion-based steganography divides into two methodological classes based on payload modality. Image-to-Image (I2I) approaches transform a secret image into a stego image via DDIM inversion, with the transformation gated by shared keys or prompts, requiring a cover-secret image pair but enabling high-capacity visual data embedding. Text-to-Image (T2I) approaches encode secret bit sequences directly into the latent diffusion trajectory, requiring no cover image and carrying no risk of key-content correlation. This makes them suitable for pure message concealment rather than image hiding. Whereas I2I approaches are conditioned on a source image, they remain synthesis-based under our taxonomy: the stego image is produced entirely through the diffusion denoising process rather than through direct pixel-level modification of the cover. The source image serves as semantic conditioning rather than as a carrier being perturbed, making the operation fundamentally generative rather than modificative.

Image-to-Image (I2I) Steganography

The foundational I2I framework was established by Yu et al. [19] with CRoSS, the pioneer zero-shot diffusion steganography method exploiting DDIM’s invertibility. By encoding secrets into the noise trajectory during reverse diffusion, CRoSS enables extraction through deterministic forward inversion without task-specific training. However, heavily conditioning generation on large image payloads introduced a fidelity bottleneck, with PSNR dropping to approximately 24 dB under high-capacity loads.

To address this fidelity degradation, Lu et al. [20] proposed VINE, employing frequency-aware training with a frozen SDXL-Turbo backbone. By learning an auxiliary embedding network operating in the frequency domain while keeping the diffusion model fixed, VINE preserves host semantic structure while achieving higher fidelity than CRoSS. However, the frozen backbone limits adaptability to image domains outside the model’s training distribution.

Extending controllability, Jiang et al. [156] introduced I2IStega, applying latent-space scrambling to ensure the stego image strictly adheres to user-provided text prompts rather than leaking the secret image’s geometry. This addresses cases where the embedded secret semantically conflicts with the desired output (e.g., hiding a landscape while prompting for portraits). Although latent scrambling ensures prompt adherence, achieving higher steganographic security requires finer control over denoising timesteps where high-frequency textures are generated.

To reduce computational overhead, Tang et al. [157] integrated ElGamal elliptic curve cryptography into the generative pipeline for anime-style I2I synthesis, minimizing the overhead associated with high-capacity embedding while maintaining semantic quality.

Text-to-Image (T2I) Steganography

Unlike I2I methods that hide images within images, T2I approaches encode binary message sequences directly into the diffusion latent space, generating stego images from scratch without requiring a cover. This eliminates visual correlation risks but shifts the challenge to ensuring the generated carrier appears natural while encoding arbitrary bit patterns. A fundamental security concern in prompt-based T2I steganography is key leakage through text prompt inference. If an adversary infers the prompt used for generation, they may reverse-engineer the embedding process or detect hidden data by analyzing prompt-content mismatches. Zhang et al. [158] proposed BUStega to eliminate this vulnerability. Rather than using manually crafted text prompts as keys, BUStega generates semantic keys automatically via BLIP [159] vision-language encoding and TF-IDF distillation, removing the correlation between prompt content and secret semantics. Saliency masks generated by U^2^-Net [160] guide embedding selectively within visually salient regions, balancing imperceptibility with precision. However, the framework still requires secure key transmission, and the additional U^2^-Net inference introduces computational overhead.

Addressing temporal control, Han et al. [161] introduced ImSA, which embeds hyperchaotic-encrypted bit sequences into the high-frequency DWT coefficients of intermediate latent variables during specific denoising timesteps. This approach exploits the hierarchical nature of diffusion: early timesteps dictate global semantic structure, and later timesteps govern fine-grained textures. By targeting later stages, ImSA integrates information into statistical regularities more effectively, enhancing undetectability against frequency-domain steganalysis.

Proactive AIGC Watermarking and Model Provenance

Whereas steganography focuses on covertness, generative watermarking prioritizes provenance, ensuring that AIGC can be traced back to its source. The most direct approach involves manipulating the initialization of the diffusion process.

Wen et al. [21] introduced Tree-Ring Watermarking, which embeds structured Fourier-domain patterns into the initial Gaussian noise vector. However, modifying the initial noise can cause unpredictable semantic shifts in the final output, as the watermark interacts with the diffusion model’s learned prior in non-linear ways.

To avoid perturbing the generative process, Fernandez et al. [22] proposed The Stable Signature, which fine-tunes only the VAE decoder of a Latent Diffusion Model but leaves the diffusion process itself entirely unchanged. By conditioning the decoder on a fixed binary signature during fine-tuning, every image the model generates inherently carries an invisible watermark. This structurally reduces susceptibility to post-generation removal attacks, as the signature is embedded in the decoder weights themselves rather than applied as a post hoc perturbation.

Subsequent research moved embedding into the sampling trajectory to improve robustness. Huang et al. [162] proposed ROBIN, which delays watermark injection until an intermediate diffusion state, using adversarial optimization to generate a “hiding prompt” that conceals the mark more naturally within semantic features. Complementing this, Lei et al. [163] developed DiffuseTrace, which tracks the full diffusion trajectory to ensure provenance remains detectable even after complex post-generation manipulations (e.g., inpainting, style transfer).

Targeting the latent domain, Meng et al. [164] performed watermark injection entirely within the compressed latent space of LDMs. By operating in latent rather than pixel space, this approach structurally decouples imperceptibility from robustness, though watermark fidelity depends critically on the VAE’s reconstruction quality.

Beyond academic prototypes, the generative landscape has increasingly expanded toward commercial foundation models and scalable generative architectures. In industrial production, proprietary frameworks such as Google’s SynthID [165] have demonstrated the feasibility of internet-scale watermarking across billions of images and video frames. Rather than relying on fragile single-band embeddings, such production systems apply multi-scale imperceptible signatures that remain verifiable under low false positive rates (FPR<0.1%) even after common compression, cropping, and downstream social media transformations. Nonetheless, the authors acknowledge notable operational trade-offs: as a post hoc framework, it introduces non-zero encoding latency and subtle lossy distortion to rendered outputs. Furthermore, maintaining imperceptibility remains challenging on unstructured corner-case content (such as flat graphics or monochrome images), and public detection remains susceptible to adversarial removal if model weights are exposed.

Cross-Modal Extension

Extending diffusion steganography to cross-modal scenarios, Xu et al. [166] introduced StegaFusion, unifying steganography across image, audio, and text modalities. By reframing data hiding as deterministic inpainting guided by shared seeds, StegaFusion enables plug-and-play deployment across diverse digital ecosystems without additional training. However, the shared-seed dependency limits flexibility in adversarial scenarios where seed security cannot be guaranteed.

#### 3.2.3. Summary and Analysis of Synthesis-Based Methods

Synthesis-based approaches generate the carrier image as part of the hiding process rather than embedding data into pre-existing content. This offers the potential for visually natural carriers and avoids some artifacts associated with post hoc embedding. However, visual naturalness does not necessarily imply statistical security: generative models may introduce detectable spectral or statistical fingerprints, while secret conditioning can shift the output distribution and create detectable signatures. Consequently, synthesis-based methods should be evaluated not only through perceptual quality but also against steganalysis targeting generative fingerprints and conditioning-induced distribution shifts. Table 5 summarizes the evolution from early GAN-based generative steganography to modern diffusion-based frameworks, highlighting their core mechanisms and inherent constraints.

Early GAN-based generative steganography (Volkhonskiy et al. [24], S-CycleGAN) demonstrated the viability of generating carriers conditioned on secret payloads, achieving strong statistical security. However, GANs suffered from mode collapse, training instability, and inability to guarantee semantic coherence under heavy payload conditioning. Diffusion models addressed these problems through iterative denoising, achieving superior visual quality and training stability. Methods like VINE, I2IStega, and BUStega demonstrated robust message recovery after geometric attacks, whereas proactive watermarking approaches (Tree-Ring, Stable Signature, ROBIN) successfully embedded provenance signals surviving post-generation edits. However, three fundamental limitations constrain deployment.

First, stochastic generation variability: Diffusion models generate images via stochastic sampling from learned distributions. Even with deterministic samplers like DDIM, identical text prompts produce entirely different visual carriers unless the initial latent noise seed is strictly shared and fixed. This inherent variability is a direct consequence of the generative paradigm: the model samples from $p_{\theta }\left(x|c\right)$ rather than deterministically transforming fixed inputs. For applications requiring reproducible outputs (forensic authentication, medical archiving), this stochasticity fundamentally limits deployment to scenarios where on-demand generation is acceptable.

Second, prohibitive computational cost: Diffusion requires iterative multi-step denoising, with each step involving a full U-Net forward pass. For 512×512 images at 50 steps, this yields 50× the computational cost of single-pass CNN encoders. Reported inference times (several seconds per image [21]) are two orders of magnitude slower than single-pass methods (10–50 ms). Although distilled models reduce this to 1–2 s, they often sacrifice quality and robustness. This computational burden makes synthesis-based methods impractical for real-time authentication or resource-constrained deployment scenarios.

Finally, capacity-fidelity trade-off: I2I methods embedding full secret images often exhibit semantic drift or color inconsistencies under high-capacity loads. The diffusion prior $p_{\theta }\left(x\right)$ conflicts with payload conditioning, forcing sampling from low-probability regions of the learned distribution. Although VINE (frequency-aware training), I2IStega (latent scrambling), and StegaFusion (deterministic inpainting) mitigate this through specialized strategies, they introduce additional complexity without fully resolving the underlying tension between generation fidelity and embedding capacity.

In summary, synthesis-based methods excel at statistical imperceptibility and proactive watermarking by design, operating within the natural image manifold by embedding data directly into the generative process rather than applying post hoc pixel modifications. However, their deployment is constrained by stochastic generation variability, computational cost two orders of magnitude higher than single-pass methods, and persistent capacity-fidelity trade-offs when conditioning on large payloads. These limitations define the operational scope: synthesis is viable for AIGC provenance tracking and covert communication scenarios where on-demand carrier generation is acceptable, whereas applications requiring reproducible outputs or real-time processing necessitate alternative paradigms.

**Table 5:** Milestone synthesis-based architectures in image data hiding (2017–2026). Type: S (Steganography), W (Watermarking). Arch: GAN (Adversarial), DPM (Diffusion Probabilistic Model), LDM (Latent Diffusion Model).

Ref.	Year	Framework	Arch.	Type	Core Mechanism	Key Contribution	Main Limitation
GAN-Based Generative Steganography							
[24]	2017	Volkhonskiy et al.	GAN	S	Generative Carrier	First synthesis-based approach using DCGAN to generate statistically secure carriers.	Low capacity; prioritizes security over embedding volume.
[25]	2019	S-CycleGAN	GAN	S	Cycle Consistency	Embeds messages during unpaired image-to-image translation via adversarial loss.	Cycle consistency constraint fundamentally limits embedding capacity.
Diffusion-Based Generative Steganography							
[19]	2023	CRoSS	DPM	S	Prompt Inversion	Pioneer zero-shot diffusion steganography via invertible denoising guidance.	Fidelity bottleneck under high-capacity loads.
[20]	2024	VINE	DPM	S	Freq.-Aware Training	Preserves host semantics via frozen SDXL-Turbo with frequency-domain guidance.	Frozen backbone limits adaptability to new image domains.
[156]	2026	I2IStega	DPM	S	Latent Scrambling	Decouples secret geometry from generation via latent-space scrambling.	Scrambling may degrade prompt adherence under high payload conditions.
[166]	2026	StegaFusion	DPM	S	Deterministic Inpainting	Plug-and-play cross-modal hiding across image, audio, and text without retraining.	Shared-seed dependency limits flexibility in adversarial deployment.
Proactive AIGC Watermarking							
[21]	2023	Tree-Ring	DPM	W	Noise Initialization	Embeds structured pattern into initial Gaussian noise for provenance tracking.	Initial noise modification causes unpredictable semantic shifts.
[162]	2024	ROBIN	DPM	W	Trajectory Embedding	Delays embedding to intermediate diffusion state for higher visual fidelity.	Adversarial prompt optimization significantly increases computational overhead.
[163]	2024	DiffuseTrace	DPM	W	Trajectory Tracking	Tracks full diffusion trajectory to ensure provenance survives post-generation edits.	Full trajectory storage introduces significant memory overhead.
[164]	2024	Meng et al.	LDM	W	Latent Injection	Watermark intrinsic to compressed LDM representation via latent-space injection.	Watermark fidelity depends on VAE reconstruction quality.

### 3.3. Logic-Based Hiding

Logic-based methods represent the most conservative end of the data hiding spectrum: no image is modified, and no image is generated. Instead, ownership or provenance is established by extracting robust, invariant features from the original content and constructing a separate verification record. This makes logic-based approaches uniquely suited to sensitive domains such as medical imaging, where pixel-level integrity is a hard constraint. This paradigm encompasses two distinct mechanisms: zero-watermarking, which constructs a cryptographic verification key from deep image features without touching the source, and coverless steganography, which encodes a message by selecting an existing unmodified image whose semantic content maps to the payload.

From the extraction requirement perspective introduced in Section 2, logic-based methods occupy a distinct position. Zero-watermarking is a non-blind scheme: verification requires the stored key constructed from the original image’s features, making the original content implicitly necessary for authentication. Coverless steganography, however, spans both semi-blind and blind categories depending on the specific embedding mechanism. Methods that rely on a shared feature-to-message mapping database or index (e.g., mapping specific object labels to bit sequences) are semi-blind, as the receiver requires this auxiliary database to decode the payload. Conversely, more advanced coverless methods are fully blind: the receiver reconstructs the message solely by applying a shared, deterministic feature extraction network to the retrieved image, requiring no index database, original image, or auxiliary secret key.

Early zero-watermarking schemes relied on frequency-domain transforms (DCT, DWT) and handcrafted feature descriptors, but these methods struggled against geometric attacks due to their inability to capture high-level semantic content [26]. The integration of deep learning has fundamentally transformed this landscape, progressing through distinct paradigms: pretrained feature extraction, contrastive learning, reconstructive architectures, attention-based transformers, and unsupervised adversarial training.

#### 3.3.1. Zero-Watermarking Methods

Zero-watermarking differs fundamentally from traditional embedding. Rather than altering the cover image to hide a payload, it operates by constructing a logical link between the image and the owner. The process typically involves extracting robust, invariant features from the original content and logically combining them with the watermark to create a separate verification key [167]. The original data remains unaltered, and ownership is verified by re-extracting features and comparing them with the stored key. This approach is particularly critical in fields like medical imaging, where even minor pixel modifications can compromise diagnostic accuracy [168].

Pretrained CNNs as Feature Extractors

The first generation of deep learning-based zero-watermarking treated pretrained classification networks (VGG, ResNet, Inception) as frozen feature extractors. Unlike handcrafted algorithms, these CNNs capture hierarchical semantic information that is inherently more robust to content-preserving manipulations. A standard pipeline involves three stages: extracting deep feature maps from a pretrained network, compressing them into a binary logical sequence via perceptual hashing, and fusing this sequence with an encrypted watermark.

Sheng et al. [169] established a baseline by combining ResNet50 features with DCT and perceptual hashing, generating the verification key through XOR operations between the binary feature vector and watermark payload. Fan et al. [170] applied Inception V3 and DCT to medical images, extending the framework to support multiple watermark types secured by chaotic encryption. Nawaz et al. [171,172] demonstrated that hybridizing deep features from ResNet101 or MobileNetV2 with frequency transforms (DWT, DCT, DTCWT) significantly improves resistance to geometric distortions compared to using deep features alone. Abdel-Aziz et al. [173] refined this fusion-domain strategy by employing Stationary Wavelet Transform alongside pretrained DarkNet-53, leveraging SWT’s shift-invariance to preserve local details often lost in standard downsampling.

The VGG19 architecture remains popular for medical zero-watermarking due to its strong texture extraction capabilities. Xiang et al. [174] utilized VGG19 to extract Gram matrix features capturing global style correlations, secured by Lorenz hyperchaotic encryption. Han et al. [175] and Gharib et al. [176] proposed hybrid schemes fusing VGG19 semantic features with localized descriptors (such as Local Binary Patterns or DFT) to ensure survival under ripple distortions and shearing.

However, relying on pretrained classifiers trained for object recognition rather than watermark robustness inherently limits their effectiveness. These networks learn features optimized for semantic categorization, not geometric invariance or attack resistance. The frozen weights prevent adaptation to watermarking-specific requirements, motivating a shift toward learnable feature extraction.

Contrastive Learning and Reconstructive Architectures

To address the limitations of frozen pretrained models, recent works have adopted contrastive learning and reconstructive training to explicitly optimize encoders for robustness. Rather than treating the network as a fixed feature extractor, these methods train the backbone specifically for zero-watermarking through self-supervised objectives.

Tong et al. [177] proposed ConZWNet, a two-stage framework employing contrastive learning on weak-strong data augmentations. By training the ConvNeXt backbone to minimize the distance between an image and its attacked versions, ConZWNet produces a watermark-aware feature space significantly more robust to tampering than standard pretrained features. This approach represents a fundamental shift from passive feature reuse to active feature learning optimized for attack resistance.

Xu et al. [178] introduced a Stacked Sparse Autoencoder using greedy layer-wise training with proportional conjugate gradient optimization and sparsity penalties. By enforcing strict sparsity constraints via KL divergence during encoding, the model learns compact representations that preserve the input’s intrinsic topology, producing features robust to geometric desynchronization while retaining local detail.

Transformer-Based Global Feature Extraction

Extending beyond local convolutional features, recent works have adopted ViTs to capture long-range dependencies across the entire image plane. By leveraging self-attention mechanisms, these methods achieve robustness against geometric attacks that desynchronize local features.

Li et al. [179] proposed a dual-branch architecture combining CNN local feature extraction with Swin Transformer global modeling, producing a 4096-dimensional feature vector that fuses both spatial hierarchies. The CNN branch captures fine-grained texture details, and the Swin Transformer branch models long-range spatial relationships resistant to cropping and scaling. This hybrid approach achieves superior robustness compared to single-branch CNN architectures, though at increased computational cost.

Building on this foundation, the same research team later advanced the approach by integrating Spatially-Coherent Self-Attention (SCSA) with FocalNet [180]. Unlike standard ViTs that process images as independent patch sequences, FocalNet employs hierarchical focal modulation to capture multi-scale global structure. This refinement enables the extraction of features robust to extreme geometric transformations, achieving NC above 0.90 under 50% cropping and 45-degree rotation.

Unsupervised Adversarial Learning

The most recent shift involves unsupervised adversarial training through generative adversarial networks, which fundamentally differs from pretrained, contrastive, and transformer approaches. Rather than relying on supervised classification objectives or pairwise similarity metrics, GANs learn robust features through adversarial dynamics between generator and discriminator networks.

Zhang et al. [26] proposed a zero-watermarking scheme based on a residual-based generative adversarial network (Res-GAN). Unlike classification networks that require labeled data, the Res-GAN employs unsupervised adversarial learning to extract robust features via end-to-end feature extraction. The generator integrates encoder-residual and decoder-residual modules with skip connections to compress input images into compact feature representations while alleviating gradient vanishing. During training, the generator continually adapts to the discriminator’s evolving judgment criteria, forcing it to learn increasingly robust feature representations that preserve essential image information under reconstruction constraints. The method maintains normalized correlation values above 0.84 across rotation, translation, and cropping scenarios. This approach represents a departure from supervised feature learning, demonstrating that fully data-driven adversarial training can produce robust features without manual annotation or task-specific supervision.

#### 3.3.2. Coverless Steganography

Coverless steganography eliminates the need for carrier modification by selecting pre-existing images whose intrinsic features naturally encode the secret message. Unlike modification-based methods that perturb pixel values, coverless approaches establish a deterministic mapping between image features (extracted via object detection, pose estimation, or deep hashing) and binary message segments. The sender queries a pre-constructed database to retrieve images whose feature hashes match the secret data, whereas the receiver applies the same feature extractor to reconstruct the message. This paradigm bypasses pixel-level steganalysis, as no statistical anomalies are introduced during transmission. However, the approach is fundamentally constrained by database size: achieving sufficient hash diversity for arbitrary message encoding necessitates databases containing thousands of images, and robustness to geometric attacks remains dependent on the stability of the chosen feature representation.

Object Detection-Based Mapping

Early coverless methods relied on object detection networks to map image semantics to binary sequences. Zhou et al. [181] introduced Faster R-CNN to detect objects within images, constructing a label-based mapping table where each detected object category corresponds to a fixed binary code. This object-label mapping paradigm has been extended in subsequent works [182,183], with Luo et al. [182] encoding positional relationships between multiple detected objects to generate longer hash sequences. However, these detection-based approaches remain vulnerable to geometric attacks that disrupt bounding box coordinates and exhibit high capacity variance (ranging from 6 to 24 bits per image depending on object count), with performance degrading sharply under small-object occlusion and detection failures.

Addressing database scalability, Liu et al. [184] proposed a YOLO-based dynamic sequence-matching method designed to minimize database requirements. By scrambling object detection labels through chaotic mapping, the method reduced the required database size to approximately 200 images while achieving 19 bits per image capacity and 80% average recovery under geometric attacks. However, the reliance on object detection frameworks introduced computational overhead during database construction, and performance remained sensitive to domain shift.

Pose Estimation and Deep Hashing

Recognizing that object-level features exhibit limited invariance to geometric transformations, Tan et al. [185] shifted to human pose estimation as a more stable semantic representation. The method employs HRNet to extract 17 anatomical keypoints from images of people, mapping relative spatial relationships between pose points to hash sequences rather than absolute pixel coordinates. By leveraging high-level geometric invariants, the approach achieved 92% average robustness across multiple attack types. However, the approach remains constrained to human-centric image databases and exhibited reduced robustness under extreme noise.

Transformer-Based Semantic Encoding

Extending coverless methods to Transformer architectures, Gautam et al. [186] employed ViTs for global semantic feature extraction. By leveraging self-attention mechanisms across the entire image, the method achieved 20-bit capacity per image with improved robustness to geometric attacks compared to CNN-based object detection. However, performance degraded on non-natural images, and the method remains dependent on pretrained models, limiting adaptability to domain-specific databases.

#### 3.3.3. Summary and Analysis of Logic-Based Methods

Logic-based approaches fundamentally differ from modification and synthesis paradigms by avoiding carrier alteration entirely. Table 6 summarizes the evolution of zero-watermarking and coverless steganography, highlighting the architectural progression from pretrained CNNs to contrastive learning to Transformers to adversarial training.

Zero-watermarking methods prioritize pixel integrity by extracting robust features and constructing external verification keys. The architectural progression from frozen pretrained extractors (VGG19, ResNet50) to learnable contrastive frameworks (ConZWNet) to global Transformer modeling (FocalNet, Swin) to unsupervised adversarial training (Res-GAN) reflects a shift from passive feature reuse to active robustness optimization. However, zero-watermarking remains constrained by binary verification: authentication is an all-or-nothing decision based on correlation thresholds, providing no mechanism for partial recovery or error correction under extreme attacks.

Coverless steganography achieves inherent steganalysis resistance by mapping semantic features to message bits without carrier modification. The evolution from object detection (Faster R-CNN, YOLO) to pose estimation (HRNet) to Transformer semantic encoding (ViT) demonstrates the field’s progression toward more stable geometric invariants. However, coverless methods suffer from a fundamental database bottleneck: achieving arbitrary message encoding requires exponentially large databases, as each additional message bit increases the required image count. Attempts to reduce database size through adaptive hash length selection successfully minimize storage requirements but introduce information loss during extreme attacks, as shorter hash sequences provide fewer redundancy bits for error correction.

Both paradigms exhibit catastrophic failure modes under extreme geometric transformations. Zero-watermarking achieves strong performance under moderate attacks but degrades sharply beyond certain thresholds, even for Transformer-based methods. Coverless steganography exhibits similar brittleness: semantic features become unstable under extreme noise, rotation, or scaling, where even pose estimation-based methods experience significantly reduced recovery rates.

In summary, logic-based methods excel at zero-distortion verification and absolute steganalysis resistance but remain constrained by binary authentication decisions (zero-watermarking) and exponential database requirements (coverless steganography). These limitations define clear deployment boundaries: zero-watermarking is viable for regulatory compliance scenarios requiring pixel-perfect integrity (medical imaging, forensic evidence), whereas coverless steganography is viable for low-bandwidth covert communication where imperceptibility outweighs capacity constraints and database construction is feasible.

### 3.4. Paradigm Comparison and Selection Criteria

Selecting an appropriate paradigm depends strictly on operational requirements rather than architectural preference alone. Modification-based methods alter existing images, offering strong resilience to transmission distortions at the expense of subtle statistical traces. Synthesis-based methods generate carriers during embedding, enabling proactive AIGC watermarking at the cost of heavy generative compute. Logic-based methods extract invariant features without modifying or synthesizing content, guaranteeing absolute pixel integrity for sensitive domains. Table 7 summarizes these operational constraints into a direct decision framework.

**Table 6:** Milestone logic-based architectures in image data hiding (2019–2026). Type: W (Watermarking), S (Steganography). Arch: CNN (ConvNet), ViT (Transformer), AE (Autoencoder), GAN (Generative Adversarial Network).

Ref.	Year	Framework	Arch.	Type	Core Mechanism	Key Contribution	Main Limitation
Zero-Watermarking							
[169]	2023	Sheng et al.	CNN	W	ResNet50 + DCT Hash	Established the standard three-stage deep feature zero-watermarking pipeline.	ResNet50 optimized for classification, not watermark robustness.
[172]	2024	Nawaz et al.	CNN	W	Deep + Freq. Fusion	Hybridizing ResNet/MobileNet with DWT/DCT improves geometric robustness.	Increased pipeline complexity raises computational cost.
[177]	2025	ConZWNet	CNN	W	Contrastive Learning	Trains ConvNeXt explicitly for robustness via weak-strong augmentation pairs.	Requires carefully designed augmentation pairs; sensitive to pair selection.
[178]	2026	Xu et al.	AE	W	Sparse Autoencoder	KL-penalized sparsity produces topology-preserving geometry-robust features.	Greedy layer-wise training significantly slower than end-to-end approaches.
[180]	2026	Li et al.	ViT	W	SCSA + FocalNet	Long-range attention captures global structure resistant to cropping and scaling.	Attention overhead limits real-time deployment on resource-constrained devices.
[26]	2026	Zhang et al.	GAN	W	Res-GAN Adversarial	Unsupervised end-to-end feature learning without labels via adversarial training.	Adversarial training introduces longer convergence time and stability concerns.
Coverless Steganography							
[181]	2019	Zhou et al.	CNN	S	Object Detection Mapping	Pioneer use of Faster R-CNN to map object classes to covert bit sequences.	Capacity strictly limited by detectable object classes per image.
[182]	2021	Luo et al.	CNN	S	Multi-Object Mapping	Positional relationships between detected objects generate longer hash sequences.	High capacity variance (6–24 bits) depending on object count per image.
[184]	2024	Liu et al.	CNN	S	YOLO + Confusion Map	Dynamic label scrambling reduces database to 200 images with 19-bit capacity.	Significantly less robust to noise attacks than modification-based methods.
[185]	2024	Tan et al.	CNN	S	HRNet Pose Estimation	Geometric invariants from 17 keypoints achieve 92% robustness to attacks.	Constrained to human-centric databases; degrades under extreme noise.
[186]	2025	Gautam et al.	ViT	S	Vision Transformer	20-bit capacity per image via self-attention global context modeling.	Performance degrades on non-natural images; pretrained model dependency.

**Table 7:** Paradigm selection by deployment requirements.

Requirement	Modification	Synthesis	Logic
Protect existing images	Yes. Embeds into legacy content	No. Requires generation from scratch	Yes. Extracts features without modification
Preserve exact pixels	No. Introduces perturbations	No. Generates new carrier	Yes. Zero modification guaranteed
Survive JPEG/crop/noise	Yes. Trained for robustness	No. Fragile to compression	Partial. Depends on feature stability
Resist steganalysis	Partial. Detectable by SRNet	Yes. Statistically natural	Yes. No modifications to detect
Watermark AIGC at generation	Partial. Post-generation only	Yes. Embedded in diffusion process	No. Cannot intervene in generation

Having reviewed the three operational paradigms, we summarize their reported performance, computational demands, and practical trade-offs in Table 8. Rather than establishing a direct ranking across incompatible experimental setups, this compilation documents each architecture’s payload capacity and visual fidelity alongside its reported hardware footprint.

The reported metrics highlight the core trade-offs across paradigms: high-capacity invertible networks achieve high visual fidelity in distortion-free channels but degrade sharply under lossy compression, whereas diffusion-based models resist complex generative editing at the cost of multi-billion parameter backbones and multi-step execution overhead. Furthermore, the prevalence of unrecorded FLOPs, memory footprints, and runtimes underscores that computational feasibility remains an overlooked constraint in the literature.

### 3.5. Practical Application Scenarios

The practical suitability of deep-learning-based image data hiding depends strongly on the requirements of the target application. While the three paradigms discussed above provide different technical capabilities, their practical deployment is determined by factors such as robustness, pixel-level integrity, authentication requirements, computational cost, and the nature of the content being protected.

For copyright protection and content distribution, the primary requirement is persistent identification of existing content after operations such as compression, resizing, and format conversion. Modification-based watermarking is therefore particularly suitable for applications involving stock photography, digital art, and media distribution, where robustness and imperceptibility are important [88,111,152]. For generative content, synthesis-based watermarking provides an alternative by incorporating provenance or ownership information during the content-generation process [21,22,165].

Medical imaging imposes stricter integrity requirements because modifications to diagnostic images may be undesirable even when they are visually imperceptible [1,15]. Zero-watermarking is therefore attractive for authentication without altering the original image [170,174,177], while reversible and invertible approaches can provide auxiliary-data embedding with exact recovery of the original content [122,124,135]. Similar requirements arise in applications involving sensitive image records where preservation of the original pixel values is essential [145].

Digital forensics and content authentication require reliable detection of unauthorized manipulation in addition to ownership verification. Applications in journalism, legal evidence management, and online content verification may involve splicing, inpainting, object removal, or AI-generated modifications [2]. Semi-fragile watermarking and localization-oriented deep-learning approaches are consequently relevant because they can support both authentication and identification of manipulated regions [91,131].

Industrial, IoT, and edge deployments introduce additional constraints related to computational complexity, memory consumption, inference latency, and hardware compatibility. Lightweight convolutional architectures are therefore more suitable for resource-constrained environments [120,150], whereas larger Transformer and generative architectures may require compression or other optimization before deployment [20,151]. In secure or covert communication, by contrast, the primary objective is to conceal the existence of embedded information, making capacity, imperceptibility, and resistance to steganalysis more important than robustness to severe image transformations [19,108,126,185].

These scenarios illustrate that no single paradigm is optimal across all deployment environments. Modification-based methods are well suited to protecting existing content, synthesis-based methods are particularly relevant to generated content, and logic-based methods are advantageous when pixel-level integrity must be preserved. Practical system design should therefore select the underlying paradigm according to the operational requirements and threat model of the intended application.

## 4. Open Challenges and Future Directions

The review presented in Section 3 reveals that progress in deep learning-based image data hiding has been largely architectural. Each new paradigm or sub-architecture addressed a specific limitation of its predecessor, yet several fundamental barriers to real-world deployment remain unresolved and cut across all three paradigms. This section identifies four such challenges and proposes concrete research directions for addressing each. A final subsection then considers emerging trends driven by foundation models that extend these challenges and introduce new research opportunities and security considerations.

### 4.1. Benchmarking Fragmentation

The most fundamental barrier to addressing the subsequent challenges is the absence of standardized evaluation infrastructure. Without consistent benchmarking protocols, robustness claims, generalization performance, and computational feasibility cannot be meaningfully compared across methods or paradigms.

Across the reviewed literature, methods are evaluated on different datasets, at different resolutions, under different attack configurations, and at different JPEG quality factors. A higher PSNR in one paper may simply reflect a lower embedding capacity or a weaker attack suite rather than a genuinely superior architecture, yet such comparisons are routinely drawn. This problem affects all three paradigms: modification-based methods are compared using incompatible JPEG quality factors and payload sizes; synthesis-based methods report FID on different generative backbones and datasets; logic-based methods evaluate feature stability under non-standardized attack sequences.

Research Directions. The primary barrier is not a lack of standards but inconsistent reporting and lack of reproducibility. The community would benefit from adopting standardized attack configurations (JPEG QF 50/70/90, 25% crop, Gaussian noise σ = 10/25) when feasible, or at minimum report complete evaluation protocols (dataset versions, preprocessing steps, attack parameters) to enable replication. Releasing pretrained model weights and evaluation code alongside publications would enable independent verification of reported results and allow the community to build directly on prior work rather than reimplementing baselines from scratch.

### 4.2. Narrow and Static Robustness Evaluation

The overwhelming majority of reviewed methods are tested against a fixed set of signal-processing distortions: JPEG compression at one or two quality factors, Gaussian noise, and basic geometric transformations such as cropping and rotation. This evaluation regime does not reflect the broader threat landscape that deployed systems face.

For modification-based methods, attack suites rarely extend beyond the noise layers used during training, meaning robustness is measured against exactly the distortions the model was designed to survive. For synthesis-based methods, robustness evaluation is often absent entirely, as generative steganography papers focus on distributional imperceptibility rather than payload survivability under attack. For logic-based methods, zero-watermarking schemes are typically tested against standard image processing operations but rarely against adversarial feature perturbations that could corrupt the verification key without visibly altering the image.

More broadly, watermark security involves threats beyond removal by signal distortion. Watermark copying and forgery attacks may attempt to reproduce a valid watermark, framing attacks may cause an innocent image to appear associated with a watermark, and collusion attacks may exploit multiple differently watermarked copies to estimate or suppress the embedded signal. Generative redrawing, visual paraphrasing, and semantics-preserving transformations present a further class of threats in which the apparent content of an image is retained while the embedded information is altered or removed. These attack scenarios are rarely evaluated systematically in current deep learning-based data hiding.

Beyond adversarial threats, real transmission scenarios introduce distortion chains that fixed attack layers do not capture: social media platforms apply proprietary recompression pipelines, print-and-scan introduces nonlinear analog distortions, and screen capture introduces moiré and lens artifacts that differ substantially from simulated JPEG. Methods specifically designed for these scenarios, such as DRAW [101] for RAW sensor pipelines and RAWIW [102] for ISP-aware watermarking, demonstrate that pipeline-aware training is feasible, but systematic evaluation under real transmission chains remains absent from the broader literature.

Research Directions. Future robustness evaluation should move beyond fixed synthetic attacks toward adaptive and realistic adversarial scenarios. Attack models should explicitly define the adversary’s access and capabilities and include watermark removal, copying, forgery, framing, and collusion attacks alongside conventional signal-level distortions. Generative redrawing, visual paraphrasing, and semantics-preserving transformations should also be incorporated to evaluate whether a watermark remains secure when the image is modified at the semantic rather than pixel level. Finally, real platform distortion chains should be characterized empirically and used for evaluation and, where appropriate, training rather than relying solely on idealized JPEG approximations.

### 4.3. Domain Generalization Gap

The overwhelming majority of methods across all three paradigms are trained and evaluated exclusively on natural image datasets [74,75], where high-frequency embedding signals are naturally masked by complex visual content. Performance on smooth or domain-specific imagery is almost never reported, despite these being precisely the domains where data hiding carries the highest operational value.

This gap manifests differently across paradigms. For modification-based methods, embedding signals optimized for texture-rich natural scenes introduce perceptible artifacts in smooth medical or satellite imagery where the HVS is more sensitive to low-frequency distortions. The few domain-specific extensions in the reviewed literature, such as MCANet [141] for depth-image rendering and DRAW [101] for RAW sensor output, demonstrate that domain-aware training is necessary rather than optional, but these remain isolated cases. For logic-based methods, feature extractors pre-trained on ImageNet classification tasks capture semantic features optimized for object recognition rather than watermark robustness, and their transferability to out-of-distribution domains such as medical scans or remote sensing imagery is largely unvalidated. ConZWNet [177] partially addresses this by training the feature extractor explicitly for robustness via contrastive learning, but domain shift remains an open problem.

Research Directions. Future research should evaluate methods on diverse image types beyond natural scenes, including medical scans, satellite imagery, high-resolution photography, low-resolution thumbnails, and document images, to demonstrate whether performance generalizes or remains dataset-specific.

### 4.4. Computational Feasibility and Deployment Validation

Computational requirements vary substantially across paradigms. Modification-based methods (CNNs, GANs, INNs) typically rely on single-pass or few-pass feedforward architectures, enabling fast processing. Transformer architectures introduce higher computational overhead due to self-attention mechanisms, while synthesis-based diffusion methods require iterative multi-step denoising passes, resulting in significantly longer inference runtimes that limit their suitability for real-time applications.

Despite these differences, computational cost and operational complexity are rarely reported in a standardized manner. As documented in Table 8, only a small fraction of published studies report parameters, FLOPs, or inference speed. Furthermore, metrics such as inference latency and peak GPU memory consumption are inherently dependent on the underlying hardware setup, input resolution, batch size, software framework, and implementation details, making direct cross-study comparisons difficult and contributing to their frequent omission.

Beyond computational characterization, the gap between laboratory evaluation and real-world deployment remains largely unaddressed in the academic literature. Evaluations are predominantly conducted in controlled settings with synthetic attack simulations, and detailed documentation of performance under production conditions, such as real social media recompression pipelines or operational distribution workflows, is scarce in peer-reviewed work. Closing this gap between closed industrial systems and openly reproducible evaluation is a central challenge for the field.

Research Directions. It is necessary to report inference time, memory usage, and hardware requirements alongside accuracy metrics to enable practical deployment assessment. The community should prioritize pilot deployments in operational contexts: integrate watermarking into real content distribution platforms, and test against production recompression pipelines. Only through such real-world validation can the field demonstrate that architectural advances translate to deployable solutions.

### 4.5. Emerging Trends and Future Directions

Beyond the four infrastructure challenges discussed above, several emerging trends could extend image data hiding beyond conventional pixel-level embedding. Multimodal foundation models provide opportunities to exploit relationships between image, text, audio, and video, while semantic representations could enable hiding and decoding based on higher-level content [166]. Explainable AI could improve the interpretability of embedding decisions by identifying which image regions and learned features contribute to payload recovery through feature attribution and saliency methods [117]. Federated learning could similarly enable training across distributed and sensitive datasets without requiring centralized image collection, which is particularly relevant to privacy-sensitive applications [187].

These developments also introduce threats that are not captured by conventional signal-level attacks. Diffusion-based regeneration, semantic editing, inpainting, and other generative operations can produce visually or semantically similar images while removing embedded information [188]. Cross-modal systems introduce additional risks through manipulation of semantic relationships between modalities, while hidden information consumed by autonomous agents could influence downstream decisions or actions. These threats extend the adversarial models discussed above from signal-level manipulation toward semantic and generative attacks.

Research Directions. Future work should investigate semantic-level hiding, multimodal watermarking, cross-modal secret communication, and agent-oriented data hiding alongside explainable and privacy-preserving training. Evaluation should extend beyond conventional signal-processing attacks to include generative regeneration, semantic editing, and cross-modal transformations. Benchmarks should also consider semantic consistency, provenance, and interpretability alongside imperceptibility, payload recovery, and robustness, providing a basis for evaluating data hiding systems against increasingly capable foundation-model-based adversaries.

## 5. Conclusions

This survey has organized deep learning-based image data hiding through a three-paradigm taxonomy and systematically analyzed 103 selected papers spanning from 2017 to early 2026. The central finding is that the field has undergone two distinct transitions, each driven by structural limitations of the preceding approach, and that neither transition has resolved the fundamental trade-off between robustness, capacity, and imperceptibility.

The first transition was architectural, occurring entirely within the modification-based paradigm. CNNs introduced end-to-end learnable embedding but suffered from non-invertible information loss that fundamentally limited recovery accuracy. GANs shifted the optimization objective from pixel-wise error minimization to statistical imperceptibility via adversarial training, resolving blurring artifacts but leaving spectral traces detectable by modern steganalyzers [68] and introducing convergence instability. INNs resolved the recoverability problem through bijective mapping, enabling near-lossless cover recovery, but remain fragile to channel distortions and dependent on estimating lost high-frequency information discarded during embedding. Transformers subsequently improved geometric robustness through global self-attention, but their lack of translation-invariance inductive bias means they require substantially more training data than CNNs to generalize effectively. Each generation advanced one vertex of the robustness-capacity-imperceptibility trilemma while displacing the constraint elsewhere.

The second transition was paradigmatic. Synthesis-based methods dissolved the boundary between carrier and payload by generating the cover as part of the hiding process, enabling deep provenance integration at the cost of applicability to existing content and computational feasibility in real-time scenarios. Logic-based methods eliminated image modification entirely, exchanging embedding capacity for an absolute pixel integrity guarantee that no modification-based architecture can provide under any configuration. As Table 7 makes explicit, there is no universal solution to image data hiding, only context-specific trade-offs between what is protected, how it is protected, and at what operational cost.

The four infrastructure gaps identified in Section 4 reveal that remaining barriers are not architectural but systemic. Benchmarking fragmentation prevents meaningful comparison across methods and paradigms. Narrow robustness evaluation measures performance against idealized distortions rather than real-world attack chains. The domain generalization gap reflects training exclusively on natural images whereas operational value concentrates in medical, satellite, and document domains. Finally, although proprietary industry solutions like Google’s SynthID [165] have successfully demonstrated the commercial viability of synthesis-based AIGC watermarking at scale, open academic literature still largely suffers from computational infeasibility and an absence of deployment validation. Addressing these challenges requires a coordinated community effort to establish evaluation infrastructure, develop domain-aware architectures, and validate claims through pilot deployments in operational contexts. The field has made substantial progress in what it can hide and where it can conceal it. The next frontier is demonstrating that these capabilities hold under the constraints, distortions, and adversaries present in real-world deployment.

## Figures and Tables

**Table 1:** Comparison of our survey with existing review literature.

Ref.	Year	Domain/Scope	Coverage Range	Taxonomy/Organization
[17]	2022	Robust image watermarking	2010–2022	By embedding domain (traditional) and training stage (single-stage vs. double-stage) for DL methods
[18]	2023	Image identity protection	1995–2023	By embedding domain (spatial, transform, hybrid), then by watermarking attributes (robust/fragile, blind/non-blind)
[14]	2023	DL-based image watermarking	2017–2023	By DL role: embedder-extractor, feature transformation networks, and hybrid methods
[10]	2023	General DL data hiding	2017–2023	By architecture (CNN, GAN) and noise injection strategy
[11]	2024	DL-based image watermarking	2020–2024	By DL architecture (GAN, DNN, CNN)
[12]	2024	General DL data hiding	2017–2023	By neural network type (DNN, CNN, GAN, INN), structure model, and training strategy
[13]	2024	General DL data hiding	2018–2023	By role of NN in embedding pipeline
[15]	2026	Medical image watermarking	2004–2025	By embedding domain (spatial, frequency, transform)
[16]	2026	Image copyright protection	2018–2025	By technology layer (DW, DL, Blockchain)
Ours	2026	General DL data hiding	2017–2026	By operational paradigm (modification, synthesis, logic)

**Table 2:** Quantitative evaluation metrics used across the three operational paradigms. For standard metrics, ↑ indicates higher is better and ↓ lower is better. AUC is an exception: the ideal value is 0.5 (statistically indistinguishable from random guessing), with values approaching 1.0 indicating increasing detectability by a steganalyzer.

Metric	Formula	Symbol Key	Description
Robustness			
BER ↓	$\frac{1}{N}\sum_{i=1}^{N}\left(x_{i}⊕y_{i}\right)$	*N*: Payload length $x_{i}$: Original bit $y_{i}$: Extracted bit	Bit Error Rate Ratio of incorrectly extracted bits. Functional decoders operate in [0,0.5]; 0.0 represents perfect recovery, while 0.5 indicates random guessing.
NCC ↑	$\frac{\sum (x_{i}\cdot y_{i})}{\sqrt{\sum x_{i}^{2}\cdot \sum y_{i}^{2}}}$	x,y: Original and extracted signals	Normalized Cross-Correlation Measures structural similarity of signals. Invariant to global linear scaling of intensity. Ranges from −1 to 1 (or 0 to 1 for non-negative binary payloads).
Imperceptibility			
PSNR ↑	$10\log_{10}\;\left(\frac{L^{2}}{\mathrm{MSE}}\right)$	*L*: Max intensity (255) MSE: Mean Squared Error	Peak Signal-to-Noise Ratio Mathematical, pixel-level fidelity proxy. Calculates absolute reconstruction error; higher values indicate lower pixel-wise distortion, though it does not serve as a direct guarantee of perceptual visual quality.
SSIM ↑	$\frac{\left(2\mu_{x}\mu_{y}+C_{1}\right)\left(2\sigma_{xy}+C_{2}\right)}{\left(\mu_{x}^{2}+\mu_{y}^{2}+C_{1}\right)\left(\sigma_{x}^{2}+\sigma_{y}^{2}+C_{2}\right)}$	μ: Mean intensity σ: Variance/Covariance $C_{1,2}$: Stability constants	Structural Similarity Index Models human perception via luminance, contrast, and structure.
LPIPS ↓	$\sum_{l}{∥w_{l}⊙\left({\hat{y}}^{\;l}-{\hat{y}}_{0}^{\;l}\right)∥}_{2}^{2}$	$w_{l}$: Layer weights ${\hat{y}}^{l},{\hat{y}}_{0}^{l}$: Stego and cover feature maps	Learned Perceptual Image Patch Similarity Semantic distance in deep feature space. Critical for generative synthesis.
FID ↓	$∥\mu_{r}-\mu_{g}{∥}^{2}+\mathrm{Tr}\;\left(\Sigma_{r}+\Sigma_{g}-2\sqrt{\Sigma_{r}\Sigma_{g}}\right)$	$\mu_{r,g}$: Feature means $\Sigma_{r,g}$: Covariance matrices Tr: Matrix trace	Fréchet Inception Distance Distributional distance between generated and real image sets.
Capacity			
bpp ↑	$\frac{\left|M\right|}{H\times W}$	|M|: Total payload bits H,W: Image dimensions	Bits Per Pixel Normalized information density per carrier pixel.
Payload ↑	|M|	M: Message vector	Absolute Payload Total bit volume. Typically used for fixed-length watermarks (e.g., 64-bit ID).
Security			
AUC	$\int_{0}^{1}\mathrm{TPR}\left(\mathrm{FPR}\right)\;d\mathrm{FPR}$	TPR: True positive rate FPR: False positive rate	Area Under the ROC Curve Resistance to binary steganalysis. Unlike standard classification, the ideal value is 0.5 (chance detection), while a value of 1.0 indicates complete detectability.

**Table 8:** Comparative performance benchmark and computational reporting audit of 16 representative works (2017–2025). Values without citations are reported by the original authors; cited values indicate independent third-party evaluations. N/R denotes “Not Reported”. Inference times are provided for operational context; they depend on GPU/CPU hardware, resolution, batch size, numerical precision, and framework, and are not directly comparable across studies.

Ref.	Year	Framework	Arch.	Dataset	Payload	Quality (PSNR/SSIM)	Target Distortions & Robustness	Params (M)	FLOPs (G)	Inference (ms)
Modification-Based Paradigms										
[84]	2017	Baluja et al.	CNN	ImageNet	24.0 bpp	37.9–41.0 dB [150]	Fragile (Stego only; lossy channels out of scope)	N/R	N/R	N/R
[23]	2018	HiDDeN	GAN	COCO	30–64 bits	30.1–47.2 dB/0.88–0.96	Cropout (p=0.3), blur, dropout, JPEG QF 50	0.40 [152]	3.52 [152]	N/R
[108]	2019	SteganoGAN	GAN	DIV2K, COCO	1.0–4.4 bpp	36.3–42.1 dB/0.88–0.98	Fragile (Steganalysis: SRNet, StegExpose)	N/R	N/R	N/R
[86]	2020	ReDMark	CNN	Pascal VOC, COCO	1024 bits	37.8–41.5 dB/0.98–0.99	JPEG QF 50, Gaussian noise, cropping	N/R	N/R	N/R
[124]	2021	HiNet	INN	DIV2K, COCO	24.0 bpp	44.6–49.0 dB/∼0.99	Fragile (Reversible; lossless channels)	N/R	N/R	N/R
[88]	2021	MBRS	CNN	ImageNet, COCO	30–64 bits	27.4–39.3 dB/0.89–0.95	Real JPEG QF 50, diffusion regeneration, blur	5.80 [152]	13.36 [152]	5.3 [20]
[126]	2023	DeepMIH	INN	DIV2K, COCO	48–96 bpp	34.8–41.7 dB/0.95–0.99	Fragile (Multi-image secret recovery)	N/R	N/R	N/R
[131]	2024	EditGuard	INN	COCO, AGE-Set	64 b + 2D mask	37.6–37.8 dB/0.94–0.95	AIGC inpainting, tamper localization, JPEG	5.45	102.73	69 (242 [20])
[152]	2024	IWFormer	ViT	COCO, ImageNet	64 bits	34.8–36.2 dB	Cropping, Gaussian blur, cropout, JPEG QF 50	1.69	12.34	N/R
[150]	2025	AttenHideNet	CNN	COCO, ImageNet, CelebA	24.0 bpp	40.1–51.7 dB/0.98–0.99	Fragile (Steganalysis: SRNet, StegExpose)	1.08	13.77	18
[151]	2025	WAM	ViT	COCO, DIV2K	32 bits/ROI	38.3–38.8 dB/∼0.99	Inpainting, splicing, localized multi-watermarking	97.1	N/R	N/R
Synthesis-Based Paradigms										
[21]	2023	Tree-Ring	DPM	MS-COCO, ImageNet	0-bit pattern	FID: 25.9 (vs. 25.3 base)	JPEG QF 25–90, crop/scale, color jitter, blur	865 *	N/R	N/R
[22]	2023	StableSig	LDM	COCO, ImageNet	48 bits	FID: 19.6/30.0 dB	Cropping (10% area), inpainting, JPEG QF 50	84.0	N/R	0 gen/15 ext
[19]	2023	CRoSS	DPM	Stego260	24.0 bpp	23.8 dB/NIQE 3.04	JPEG QF 20–80, Gaussian noise, screen-shooting	>860 *	N/R	N/R
[20]	2025	VINE	DPM	OpenImages, W-Bench	100 bits	37.3–40.5 dB/∼0.99	AIGC inpainting, Instruct-Pix2Pix, regeneration	3500 *	N/R	79.5
Logic-Based Paradigms										
[185]	2024	Tan et al.	CNN	VITON-HD, DeepFashion	9–15 bits	N/A (0 distortion)	Affine & geometric transforms, JPEG, noise	28.5	N/R	N/R
[177]	2025	ConZWNet	CNN	MiniImageNet	1536-bit key	N/A (0 distortion)	Rotation (180°), severe cropping, JPEG QF 20	50.0	N/R	N/R

Notes: N/R = Not Reported. Values with citations indicate independent third-party evaluations; otherwise, values are reported by original authors. PSNR, SSIM, and FID are retained as reported and are not directly interchangeable across distinct generation vs. embedding tasks. Reported inference times are hardware-dependent and provided for operational context rather than direct cross-method ranking. * Backbone model parameter count.

## Data Availability

No new data were created or analyzed in this study. Data sharing is not applicable to this article.
